# A Bayesian network meta-analysis: evaluating the efficacy and safety of targeted therapies in metastatic or advanced radioiodine-refractory differentiated thyroid cancer

**DOI:** 10.3389/fonc.2026.1720670

**Published:** 2026-02-27

**Authors:** Pin Wang, Ling Li, Ying Liu, Yushuya Shi, Xiangyu Zhang, Jian Wu

**Affiliations:** 1 Department of General Surgery, The Third People’s Hospital of Chengdu, Chengdu, China; 2 Center of Breast and Thyroid Surgery, The Third People’s Hospital of Chengdu, Chengdu, China; 3 Department of Pathology, The Third People’s Hospital of Chengdu, Chengdu, China; 4 Department of Ultrasound, The Third People’s Hospital of Chengdu, Chengdu, China; 5 Department of Hepatobiliary and Breast/Thyroid Surgery, The Second People’s Hospital of Jintang County, Chengdu, China

**Keywords:** Bayesian network time-trend analysis, metastatic or advanced radioiodine-refractory differentiated thyroid cancer, objective response rate, progression-free survival, target therapy

## Abstract

**Background:**

Approximately 5%–10% of patients with differentiated thyroid cancer (DTC) develop resistance to radioactive iodine (RAI), leading to unsatisfactory survival rates. The optimal medication for advanced or metastatic RAI-resistant differentiated thyroid cancer (RAIR-DTC) remains unclear.

**Methods:**

We conducted a Bayesian network meta-analysis based on a systematic search of six electronic databases. The primary outcome was progression-free survival (PFS); secondary outcomes included overall survival (OS), objective response rate (ORR), and grade ≥3 adverse events (AEs). Hazard ratios (HRs) with 95% credible intervals (CrIs) were used for time-to-event outcomes, while odds ratios (ORs) with 95% CrIs were used for binary outcomes. A separate Bayesian network meta-analysis was performed for each endpoint.

**Results:**

Our study included 9 RCTs involving 1,760 patients with RAIR-DTC. Lenvatinib, anlotinib, apatinib, and cabozantinib all significantly improved PFS versus placebo (HRs: 3.85–5.36), with lenvatinib ranking first overall (SUCRA: 81.97%) and showing sustained benefit up to 24 months. Apatinib provided early PFS advantage but waning efficacy beyond 6–9 months. No treatment significantly improved OS, though apatinib consistently ranked highest for OS. Lenvatinib achieved the highest objective response rate (OR = 143.18; SUCRA: 82.09%). For grade ≥3 adverse events, no treatment differed significantly from placebo; however, apatinib ranked highest in safety (SUCRA = 93.16%).

**Conclusion:**

Lenvatinib demonstrates the greatest benefit in both PFS and ORR among the evaluated TKIs for RAIR-DTC, suggesting it as a potential preferred first-line option. The time-dependent efficacy patterns of other TKIs warrant further investigation.

**Systematic Review Registration:**

https://www.crd.york.ac.uk/prospero/display_record.php?ID=CRD420251089713, identifier CRD420251089713.

## 1 Introduction

Thyroid cancer has become one of the most prevalent malignant tumors in the endocrine system, with a significant increase on incidence over the past few decades (1). Differentiated thyroid cancer (DTC), which includes papillary and follicular carcinoma, accounts for over 90% of all thyroid cancer cases (2). Although many patients with papillary thyroid carcinoma (DTC) achieve a 10-year survival rate of over 90% after undergoing surgical resection, radioactive iodine (RAI) therapy, and thyroid-stimulating hormone (TSH) suppression therapy, approximately 5% to 10% of DTC patients develop refractory disease to RAI treatment, and the emergence of this refractory disease leads to the development of resistance, which significantly reduces survival rates. Patients with advanced or metastatic RAI-resistant DTC (RAIR-DTC) undergo grimmer prognosis, with a 10-year survival rate of less than 20% (3, 4). The low survival rate and high treatment difficulty of RAIR-DTC pose tough challenges to both patients and health-care providers.

The treatment landscape for RAIR-DTC has evolved over time. Historically, surgery and RAI therapy were the cornerstones of treatment for DTC. However, as resistance to RAI emerged in a subset of patients, the need for alternative therapies became urgent. In recent years, targeted therapies have emerged as promising options for patients with advanced or metastatic DTC, particularly those who are RAI-resistant. Tyrosine kinase inhibitors (TKIs) and other targeted agents have demonstrated significant anti-tumor activity in clinical trials. For example, lenvatinib was approved by the FDA for treating RAI-resistant DTC in 2015 based on the results of the SELECT trial, which showed significant advantages in progression-free survival (PFS) and objective response rate (ORR) (5). Cabozantinib also received FDA approval for this indication in 2021 based on the results of the COSMIC-311 study (6). These drugs target various molecular pathways involved in thyroid cancer progression, such as vascular endothelial growth factor receptor (VEGFR), fibroblast growth factor receptor (FGFR), and the rearranged during transfection (RET) proto-oncogene (7–9).

Despite the growing number of targeted therapies, current guidelines lack a consensus on the optimal treatment regimen for patients with advanced or metastatic DTC (10). Current treatment strategies are often based on individual clinical trials or limited comparative studies, which may not provide a comprehensive view of the relative efficacy and safety of different therapies.

Although several studies have attempted to assess the efficacy of targeted therapies through network meta-analyses, these analyses typically focused solely on direct comparisons of progression-free survival (PFS) and overall survival (OS) (11–13). They often failed to fully utilize all available data, leading to limited evaluation of efficacy and safety. This shortcoming may result in misleading conclusions or biases, thereby complicating clinical decision-making.

To better characterize how treatment effects on progression-free survival evolve over time, we conducted an exploratory Bayesian network meta-analysis incorporating follow-up duration as a continuous effect modifier. This time-trend analysis leverages available longitudinal data to assess the durability of treatment benefits, providing insights beyond fixed-time-point comparisons.

## 2 Methods

This network meta-analysis (NMA) was conducted in strict accordance with the PRISMA 2020 statement and its extension for network meta-analyses (PRISMA-NMA) (13). The protocol of this systematic review was prospectively registered in PROSPERO (CRD420251089713). A completed PRISMA 2020 checklist is provided in **Supplementary File S1** (14).

### 2.1 Search strategy

We systematically searched PubMed, Embase, Cochrane Library, Scopus, ClinicalTrials.gov, and Google Scholar from database inception to July 29, 2025. The full search strategies for each database—including controlled vocabulary (e.g., MeSH, Emtree) and free-text terms—are detailed in **Supplementary File S2**. Briefly, the search combined terms related to:

(1) disease: (“thyroid cancer” OR “differentiated thyroid cancer” OR “radioactive iodine-resistant thyroid cancer” OR “RAIR-DTC” OR “metastatic differentiated thyroid cancer” OR “advanced differentiated thyroid cancer”); and
(2) interventions: (“targeted therapy” OR “tyrosine kinase inhibitor” OR “TKI” OR “VEGFR inhibitor” OR “FGFR inhibitor” OR “RET inhibitor” OR specific drug names including “lenvatinib”, “sorafenib”, “cabozantinib”, “anlotinib”, and “apatinib”).

All statistical analyses were implemented using R version 4.4.2. The annotated R code for Bayesian network meta-analysis and time-trend modeling (based on the gemtc and rjags packages) is publicly available in **Supplementary File S3**, ensuring full reproducibility of our results.

### 2.2 Selection criteria

Included criteria:

1) RCTs with data which could be clearly and readily extracted;
2) patients aged ≥ 18 years with pathologically confirmed metastatic or advanced RAIR-DTC;
3) RCT investigated medications on endpoints of interest (i.e., OS, PFS).

Exclusion criteria:

1) non-RCTs, case reports, reviews, conference proceedings;
2) single-arm studies;
3) data with regard to interventions were mixed and could not be segregated;
4) non-English or duplicate cohorts.

### 2.3 Study enrollment and data extraction and quality assessment

After thorough retrieve on databases, Endnote X9 was utilized to remove duplicate publications. Another part of dissertations was precluded by reading the titles and abstracts, as well as full text if needed.

In this study, we extracted key data from eligible trials, including study ID, first author, region, allocations, age, gender, tumor stage, the proportion of patients previously treated with targeted therapy, regimens, and outcome measures. The quality of enrolled studies was evaluated according to Risk of Bias, 2^nd^ edition (ROB2) implementing Review Manager 5.4.

Two investigators (Pin Wang and Ling Li) independently finished the above tasks, and any discrepancy were arbitrated by a senior reviewer (Jian Wu).

### 2.4 Endpoints

The primary endpoint was PFS, defined as the time from randomization to disease progression or death from any cause.

Secondary endpoints included overall survival (OS), objective response rate (ORR), and adverse events (AEs). OS was defined as the time from randomization to death from any cause.

the proportion of patients who achieved a best overall response of either complete response (CR) or partial response (PR), as assessed by RECIST (primarily version 1.1) per each trial’s protocol. This represents a binary endpoint based on the best tumor response observed during the treatment period, irrespective of subsequent disease progression or treatment discontinuation. ORR was defined as the proportion of patients who achieved a best overall response of either complete response (CR) or partial response (PR), as assessed by RECIST (primarily version 1.1) per each trial’s protocol. This represents a binary endpoint based on the best tumor response observed during the treatment period, irrespective of subsequent disease progression or treatment discontinuation. AEs were defined as any untoward medical occurrence in a patient receiving study treatment, with particular emphasis on grade ≥3 AEs as classified according to the Common Terminology Criteria for Adverse Events (CTCAE v5.0) version employed in each trial.

### 2.5 Statistical analyses

For binary outcomes—including objective response rate (ORR) and the incidence of grade ≥3 adverse events—we extracted the number of events and total sample sizes from both the experimental and control arms of each included study. The natural logarithm of the odds ratio (lnOR) and its corresponding standard error (SElnOR) were calculated using STATA 17.0 MP. These lnOR and SE values were then imported into R version 4.4.2 and analyzed using the gemtc package to perform Bayesian network meta-analysis at prespecified timepoints. All models were fitted on the lnOR scale, and results are reported as odds ratios (ORs) with 95% credible intervals (CrIs). For efficacy outcomes such as ORR, an OR > 1 favors the treatment; for safety outcomes such as grade ≥3 adverse events, an OR < 1 indicates a more favorable safety profile.

For time-to-event outcomes—overall survival (OS) and progression-free survival (PFS)—we employed two complementary analytical approaches:

(1) Standard HR-based analysis: We directly extracted the hazard ratios (HRs) and their 95% confidence intervals as reported by the original publications. From these, we derived the natural logarithm of the HR (lnHR) and its standard error (SElnHR), which were then used as input for Bayesian network meta-analysis in the gemtc package. Results are presented as HRs with 95% CrIs. In this analysis, HRs for PFS and OS are reported as placebo (or control) versus active treatment; thus, an HR > 1 indicates a lower risk of progression or death in the treatment group and reflects a beneficial effect.
(2) Time-point-specific binary analysis: To provide clinically interpretable estimates of treatment effect at key decision-making timepoints, we further digitized the published Kaplan–Meier (KM) curves using the GetData Graph Digitizer 2.22 software. At prespecified timepoints (3, 6, 9, 12, 18, and 24 months), we extracted the number of patients at risk and the number of patients who remained event-free (i.e., alive for OS or progression-free for PFS). For each timepoint, survival status was treated as a binary outcome (e.g., “alive vs. dead at 12 months”). Based on these counts, lnORs and their standard errors were computed in STATA and subsequently analyzed using gemtc. Results from this approach are reported as time-specific ORs for survival (or PFS), enabling comparison of relative treatment efficacy at defined follow-up intervals. However, it should be noted that event statuses extracted from the same Kaplan–Meier curve at multiple timepoints (e.g., 6, 12, and 18 months) are inherently correlated within each trial. Our model treats each timepoint as an independent binary outcome due to the lack of individual patient data, which may underestimate the true uncertainty of the estimates.
(3) Time-trend modeling of treatment effects over time: To explore how treatment effects on PFS evolve across follow-up duration, we conducted a time-trend analysis by incorporating the landmark timepoint (in months) as a continuous covariate in the Bayesian network meta-analysis model. Given the limited number of studies per treatment comparison and the absence of individual patient-level data, we adopted a parsimonious model assuming a shared linear trend across all treatments. This approach assumes a shared linear trend across all treatments and estimates the change in log-hazard ratio per month. While this assumption may not capture drug-specific non-linear dynamics (e.g., early separation followed by convergence of survival curves), it provides a feasible exploratory framework to assess whether treatment effects on PFS generally increase, decrease, or remain stable over time in the context of aggregate-level evidence. The model uses weakly informative normal priors for the time coefficient (mean = 0, SD = 1) and shares the same random-effects structure as the fixed-time-point analyses (τ ∼ half-normal(0, 0.5)). Importantly, this analysis does not adjust for study-level characteristics (e.g., prior TKI use, crossover rate) and should be interpreted solely as an exploratory assessment of temporal patterns, not as an explanation of between-study heterogeneity.

Convergence of Markov Chain Monte Carlo (MCMC) simulations was rigorously assessed using multiple criteria (1): visual inspection of trace plots, density plots, and Brooks–Gelman–Rubin plots; and (2) quantitative evaluation of the potential scale reduction factor (PSRF), with values < 1.05 for all model parameters considered indicative of convergence. All models were run with four independent chains, with 20,000 burn-in and 20,000–300,000 post-burn-in iterations per chain (thinning interval = 10).

To evaluate the agreement between direct and indirect evidence in the treatment network, we assessed statistical inconsistency using two complementary approaches. First, we performed a global inconsistency assessment using the unrelated mean effects (UME) model, which relaxes the consistency assumption across all treatment comparisons. Second, we applied the node-splitting method to detect local inconsistency in specific treatment loops where both direct and indirect evidence were available. In the node-splitting analysis, the difference between direct and indirect effect estimates (the inconsistency factor) was estimated for each splittable comparison; an inconsistency was considered statistically significant if the 95% credible interval of the inconsistency factor excluded zero. All inconsistency analyses were conducted within the Bayesian framework using the gemtc package in R, with the same prior specifications and MCMC settings as the primary consistency model.

Treatment rankings were summarized using the Surface Under the Cumulative Ranking curve (SUCRA). SUCRA transforms the mean rank of each treatment into a value between 0 and 1 (or 0% to 100%), where a value of 1 indicates the treatment is always ranked first (best), and 0 indicates it is always ranked last (worst). Higher SUCRA values thus reflect better relative efficacy or safety. SUCRA was calculated using the sucra() function in the gemtc R package, based on posterior ranking distributions obtained from the Bayesian network meta-analysis model.

All network meta-analyses were conducted using a Bayesian random-effects model, with between-study heterogeneity modeled through the heterogeneity standard deviation τ (reported as sd.d). The posterior median of τ and its 95% credible interval are presented for each outcome. The primary analysis included all eligible studies Pre-specified sensitivity analyses were performed to assess robustness (1): excluding studies at high risk of bias (2); restricting to trials with direct head-to-head comparisons; and (3) re-fitting models with an alternative prior for τ. Statistical significance was determined by whether the 95% credible interval for the effect estimate (OR or HR) excluded the null value.

## 3 Results

### 3.1 Literature search and study selection

**Figure 1:**
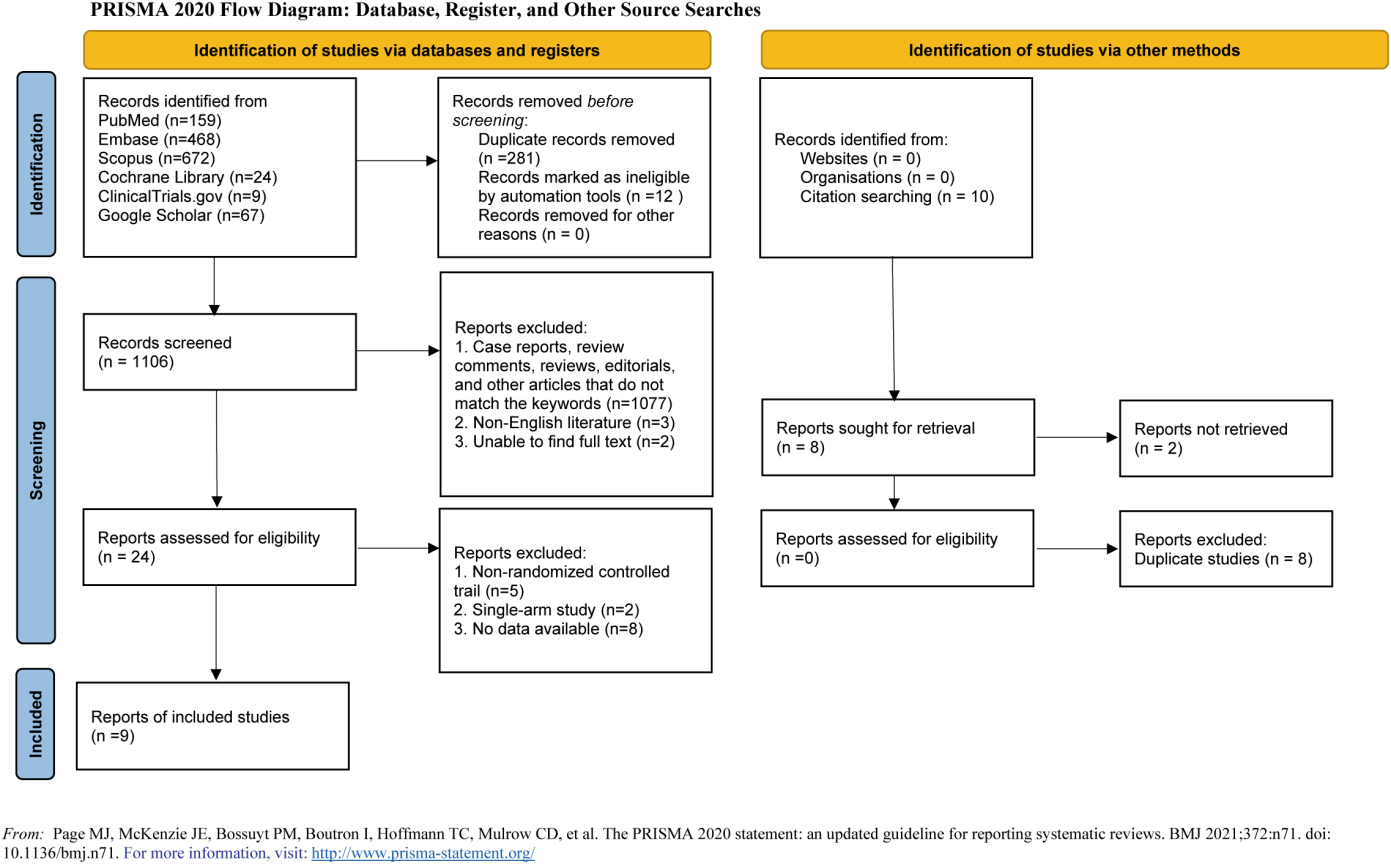
This diagram shows the PSRISMA flow diagram for study search and selection (updated in 2020). PSRISMA, Preferred Reporting Items for Systematic Reviews and Meta-Analyses.

Our preliminary search yielded 1,399 articles, eventually 9 studies published from 2012 to 2024 were included in our study (**Figure 1**) (15–23).

The enrolled studies reported 1,760 patients with metastatic or advanced RAIR-DTC receiving various medication therapies comprising sorafenib, cabozantinib, vandetanib, anlotinib, nintedanib, apatinib, and lenvatinib. The follow-up duration ranged from 6.2 to 35.9 months. The median age of the participants spanned from 56 to 66 across the studies. The overall quality of the included studies was generally satisfying. Details on the included studies and the risk-of-bias assessment are presented in **Table 1** and **Supplementary Figure S1**.

**Table 1:** Baseline demographic and clinical characteristics of the trials included in the network meta-analysis of patients diagnosed with radioiodine-refractory differentiated thyroid carcinoma.

Study (Year)	Trial ID	Median age	Region	Male/female	Follow-up (months)	Experimental Intervention	Control intervention	Experimental group (n)	Control group (n)	Outcomes	Study phase
Brose (2014) (16)	NCT00984282	Sora:63 pla:63	Europe, North America, Asia	Sora:104/103 pla:95/115	16.2	Sorafenib 400 mg, orally, twice daily	placebo	207	210	PFS, OS, ORR, grade ≥3 AEs	Phase III
Brose (2021) (20)	NCT03690388	Cabo:65 pla:66	Europe, Asia, North	Cabo:57/68 pla:28/34	6.2	Cabozantinib 60 mg, orally, once daily	placebo	125	62	PFS, OS, ORR, grade ≥3 AEs	Phase III
Brose (2024) (14)	NCT01876784	Vande:64.2 pla:63.2	USA, Europe, Asia	Vande:49/70 pla:55/64	NA	Vandetanib 300 mg, orally, once daily	placebo	119	119	PFS, OS, ORR, grade ≥3 AEs	Phase III
Chi (2023) (22)	NCT02586337	Anlo:57 pla:56	China	Anlo:33/43 pla:8/29	35.9	Anlotinib 12mg, orally, once daily	placebo	76	37	PFS, OS, ORR, grade ≥3 AEs	Phase II
Leboulleux (2012) (15)	NCT00537095	Vande:63 pla:64	Europe	Vande:39/33 pla:39/34	19.2	Vandetanib 300 mg, orally, once daily	placebo	72	73	PFS, OS, ORR, grade ≥3 AEs	Phase II
Leboulleux (2024) (21)	NCT01788982	66	Europe	Ninted:20/25 pla:11/14	21.7	Nintedanib 200 mg,orally, twice daily	placebo	45	25	PFS, OS, ORR, grade ≥3 AEs	Phase II
Lin (2021) (19)	NCT03048877	Apa:56 pla:59.5	China	Apa:19/27 pla:17/29	18.1	Apatinib 500 mg, orally, once daily	placebo	46	46	PFS, OS, ORR, grade ≥3 AEs	Phase III
Schlumberger (2015) (17)	NCT01321554	lenva:64 pla:61	Europe, North America, Asia	lenva:125/91 pla:75/56	17.4	lenvatinib 24 mg,orally, once daily	placebo	216	131	PFS, OS, ORR, grade ≥3 AEs	Phase III
Zheng (2021) (18)	NCT02966093	lenva:61 pla:60	China	lenva:57/46 pla:21/27	15.2	lenvatinib 24 mg, orally, once daily	placebo	103	48	PFS, OS, ORR, grade ≥3 AEs	Phase III

Anlotinib (Anlo), apatinib (Apa), cabozantinib (Cabo), lenvatinib (Lenva), nintedanib (Ninted), sorafenib (Sora), vandetanib (Vande), and placebo (Pla).

### 3.2 Network meta-analysis of PFS outcomes across multiple time points

**Supplementary Figure S2** presents the network plots of pairwise comparisons for PFS at each time point. Results of direct and indirect comparisons at each time point are shown in **Table 2** and **Supplementary Table S1**.

**Table 2:** Pairwise comparisons of regimens on PFS at landmark time points from Bayesian fixed-time-point network meta-analyses (reported as odds ratios with 95% credible intervals).

Time point	Control group	Pla	Lenva	Sora	Anlo	Apa	Cabo	Vande	Ninted
3 month	SUCRA(%)	11.87	66.18	40.16	63.66	87.32	69.79	31.89	29.13
	Pla	Pla	7.05 (0.86, 64.65)	2.54 (0.12, 51.08)	7.26 (0.27, 185.74)	31.46 (1.05, 1032.98)	8.71 (0.42, 182.88)	1.85 (0.24, 16.5)	1.62 (0.07, 38.15)
	Lenva	0.14 (0.02, 1.16)	Lenva	0.36 (0.01, 13.48)	1.03 (0.02, 48.07)	4.39 (0.07, 261.23)	1.22 (0.03, 50.54)	0.26 (0.01, 5.5)	0.23 (0.01, 9.94)
6 month	SUCRA(%)	7.71	79.68	35.03	83.93	70.75	67.75	31.02	24.12
	Pla	Pla	9.47 (2.75, 31.97)	2.13 (0.4, 11.52)	12.25 (1.72, 82.48)	7.22 (1.09, 47.54)	6.66 (1.13, 39.36)	1.9 (0.58, 6.5)	1.48 (0.22, 10.27)
	Lenva	0.11 (0.03, 0.36)	Lenva	0.22 (0.03, 1.83)	1.28 (0.13, 12.54)	0.77 (0.08, 7.23)	0.7 (0.08, 6.06)	0.2 (0.04, 1.15)	0.16 (0.02, 1.55)
9 month	SUCRA(%)	44.90	74.97	27.47	73.74	50.38	95.52	28.34	44.90
	Pla	Pla	13.77 (2.96, 61.95)	2.1 (0.26, 17.37)	13.83 (1.47, 128.53)	5.03 (0.55, 44.54)	142.39 (3.78, 4342.68)	2.17 (0.48, 9.85)	4 (0.33, 49.63)
	Lenva	0.07 (0.02, 0.34)	Lenva	0.15 (0.01, 2.1)	1.01 (0.06, 14.81)	0.37 (0.03, 5.21)	10.35 (0.21, 414.35)	0.16 (0.02, 1.33)	0.29 (0.02, 5.54)
12 month	SUCRA(%)	7.54	73.63	25.46	70.97	60.55	93.37	25.61	42.86
	Pla	Pla	18.03 (3.18, 116.21)	1.68 (0.14, 19)	16.9 (1.28, 225.73)	10.4 (0.79, 140.16)	138.16 (4.06, 5028.13)	1.69 (0.29, 10)	4.18 (0.19, 92.85)
	Lenva	0.06 (0.01, 0.31)	Lenva	0.09 (0, 1.79)	0.93 (0.04, 20.34)	0.57 (0.02, 13.04)	7.53 (0.14, 384.69)	0.09 (0.01, 1.15)	0.23 (0.01, 7.45)
18 month	SUCRA(%)	15.76	92.18	43.81	70.97	74.29	/	32.76	92.18
	Pla	Pla	32.1 (4.73, 274.9)	2.7 (0.2, 38.29)	10.79 (0.73, 165.36)	12.54 (0.81, 193.39)	/	1.76 (0.27, 11.55)	0.49 (0, 47.9)
	Lenva	0.03 (0, 0.21)	Lenva	0.08 (0, 2.14)	0.33 (0.01, 9.08)	0.39 (0.01, 10.6)	/	0.05 (0, 0.76)	0.01 (0, 1.97)
24 month	SUCRA(%)	13.19	89.62	30.87	58.24	58.09	/	/	/
	Pla	Pla	155.59(1.94, 12103.38)	2.15 (0.01, 635.01)	13.02 (0.04, 4174.06)	12.65 (0.04, 4364.84)	/	/	/
	Lenva	0.01 (0, 0.52)	Lenva	0.01 (0, 17.82)	0.08 (0, 116.87)	0.08 (0, 113.35)	/	/	/

Anlotinib (Anlo), apatinib (Apa), cabozantinib (Cabo), lenvatinib (Lenva), nintedanib (Ninted), sorafenib (Sora), vandetanib (Vande), and placebo (Pla). Results are based on separate Bayesian network meta-analyses conducted at each landmark time point (3, 6, 9, 12, 18, and 24 months). Missing estimates (“/”) indicate that the regimen was not evaluated at that time point in any included study.

At 3^rd^ month, apatinib significantly improved PFS compared to placebo (OR = 31.46, 95% CrI: 1.05–1032.98). Apatinib ranked first in SUCRA (87.32%), with cabozantinib ranking second (69.79%).

At 6^th^ month, lenvatinib (OR = 9.47, 95% CrI: 2.75–31.97), anlotinib (OR = 12.25, 95% CrI: 1.72–82.48), apatinib (OR = 7.22, 95% CrI: 1.09–47.54), and cabozantinib (OR = 6.66, 95% CrI: 1.13–39.36) all significantly improved PFS versus placebo. Compared with lenvatinib, no significant differences were found among lenvatinib, anlotinib, apatinib, and cabozantinib. Anlotinib ranked first in SUCRA (83.93%), with lenvatinib second (79.68%).

At 9^th^ month, vs. placebo, lenvatinib (OR = 13.77, 95% CrI: 2.96–61.95), anlotinib (OR = 13.83, 95% CrI: 1.47–128.53), and cabozantinib (OR = 142.39, 95% CrI: 3.78–4342.68) showed significantly higher PFS. When lenvatinib was the reference, no significant differences were found among these three. Cabozantinib had the highest SUCRA ranking (95.52%), followed by lenvatinib (74.97%).

At 12^th^ month, compared to placebo, lenvatinib (OR = 18.03, 95% CrI: 3.18–116.21), anlotinib (OR = 16.9, 95% CrI: 1.28–225.73), and cabozantinib (OR = 138.16, 95% CrI: 4.06–5028.13) had significantly higher PFS. Taking lenvatinib as reference, no significant differences were observed among them. Cabozantinib ranked highest in SUCRA (93.37%).

At 18^th^ month, vs. placebo, lenvatinib (OR = 32.1, 95% CrI: 4.73–274.9) had significantly higher PFS. Lenvatinib also ranked highest in SUCRA (92.18%), followed by apatinib (74.29%).

At 24^th^ month, compared to placebo, lenvatinib (OR = 155.59, 95% CrI: 1.94–12103.38) had significantly higher PFS. Lenvatinib ranked highest in SUCRA (89.62%).

Overall, from the network meta-analysis of PFS at 3, 6, 9, 12, 18, and 24 months, lenvatinib, apatinib, and cabozantinib all significantly improved PFS over placebo at multiple time points.

### 3.3 Network meta-analysis of OS outcomes across multiple time points

**Supplementary Figure S3** presents the network plots of pairwise comparisons for OS at each time point. The results of direct and indirect comparisons at each time point are shown in **Table 3** and **Supplementary Table S2**.

**Table 3:** Pairwise comparisons of regimens on OS at landmark time points from Bayesian fixed-time-point network meta-analyses (reported as odds ratios with 95% credible intervals).

Time point	Control group	Pla	Lenva	Sora	Anlo	Apa	Vande	Cabo
3 month	SUCRA (%)	22.76	36.30	33.29	73.76	68.41	49.73	65.76
	Pla	—	1.41 (0.18, 17.8)	1.24 (0.06, 28.25)	10.58 (0.18, 619.1)	7.52 (0.13, 409.04)	2.37 (0.24, 31.21)	5.13 (0.23, 111.44)
	Lenva	0.71 (0.06, 5.55)	—	0.88 (0.01, 32.57)	7.22 (0.06, 653.01)	5.11 (0.04, 444.22)	1.67 (0.05, 43.24)	3.64 (0.06, 134.3)
6 month	SUCRA (%)	25.03	55.99	28.70	73.89	75.36	28.40	62.63
	Pla	—	1.68 (0.59, 4.53)	0.99 (0.25, 3.9)	3.28 (0.39, 26.91)	3.3 (0.46, 23.19)	1.0 (0.32, 3.04)	1.99 (0.52, 7.59)
	Lenva	0.6 (0.22, 1.69)	—	0.59 (0.11, 3.35)	1.96 (0.19, 20.01)	1.98 (0.22, 18.2)	0.59 (0.13, 2.73)	1.18 (0.22, 6.53)
9 month	SUCRA (%)	21.89	62.82	29.48	62.95	85.20	39.49	48.17
	Pla	—	1.85 (0.7, 5.24)	1.04 (0.26, 4.21)	2.17 (0.34, 13.84)	4.59 (0.64, 33.79)	1.26 (0.44, 3.57)	1.47 (0.36, 5.96)
	Lenva	0.54 (0.19, 1.43)	—	0.56 (0.1, 3.08)	1.16 (0.14, 9.38)	2.48 (0.27, 22.75)	0.68 (0.15, 2.8)	0.79 (0.14, 4.28)
12 month	SUCRA (%)	21.48	56.17	41.43	70.31	88.63	25.30	46.69
	Pla	—	1.7 (0.55, 4.96)	1.32 (0.29, 5.96)	2.79 (0.42, 18.89)	6.14 (0.77, 47.37)	1.01 (0.33, 3.18)	1.46 (0.31, 6.91)
	Lenva	0.59 (0.2, 1.81)	—	0.78 (0.12, 5.17)	1.64 (0.19, 14.96)	3.63 (0.36, 36.3)	0.59 (0.13, 2.98)	0.86 (0.13, 6.05)
18 month	SUCRA (%)	0.23	0.41	0.44	0.78	0.82	0.33	/
	Pla	—	1.21 (0.49, 2.62)	1.26 (0.41, 3.8)	2.73 (0.61, 12.2)	2.92 (0.71, 11.98)	1.08 (0.46, 2.53)	/
	Lenva	0.83 (0.38, 2.03)	—	1.04 (0.27, 4.52)	2.28 (0.43, 12.97)	2.43 (0.49, 13.15)	0.9 (0.29, 3.15)	/
24 month	SUCRA (%)	27.26	23.15	51.78	81.17	80.77	35.87	/
	Pla	—	0.91 (0.36, 2.49)	1.44 (0.39, 5.31)	3.15 (0.66, 15.01)	3.06 (0.68, 13.75)	1.1 (0.28, 4.38)	/
	Lenva	1.09 (0.4, 2.77)	—	1.58 (0.3, 7.71)	3.43 (0.54, 21.19)	3.33 (0.54, 19.49)	1.2 (0.22, 6.26)	/

Anlotinib (Anlo), apatinib (Apa), cabozantinib (Cabo), lenvatinib (Lenva), sorafenib (Sora), vandetanib (Vande), and placebo (Pla). Results are based on separate Bayesian network meta-analyses conducted at each landmark time point (3, 6, 9, 12, 18, and 24 months). Missing estimates (“/”) indicate that the regimen was not evaluated at that time point in any included study.

At all-time points, no targeted therapy demonstrated a higher OS compared to placebo. In terms of SUCRA rankings, the highest scores at 3, 6, 9, 12, 18, and 24 months were achieved by anlotinib (73.76%), apatinib (75.36%), apatinib (85.20%), apatinib (88.63%), apatinib (81.84%), and anlotinib (81.17%), respectively.

### 3.4 Survival analysis of PFS, OS, ORR and AEs (grade ≥ 3)

We recorded the hazard ratios (HRs) of progression-free survival (PFS) and overall survival (OS), as well as the objective response rate (ORR) and adverse events of grade ≥3. The corresponding network plot is shown in **Supplementary Figure S4A**. The results of direct and indirect comparisons at each time point are presented in **Tables 4**, **5** and **Supplementary Tables S3**-**S6**.

**Table 4:** Matrices of pairwise comparisons of regimens on PFS and OS from Bayesian network meta-analysis and time-trend analysis (shown as hazard ratios and 95% credible intervals).

Outcomes	Methods	Control group	Pla	Lenva	Sora	Anlo	Apa	Cabo	Vande
PFS	Fixed-time-point analysis	SUCRA(%)	3.87	81.97	30.98	74.46	63.84	71.911	22.946
		Pla	Pla	5.36 (2.32, 12.6)	1.70 (0.52, 5.47)	4.78 (1.38, 16.40)	3.85 (1.11, 13.49)	4.54 (1.351, 15.185)	1.44 (0.62, 3.36)
		Lenva	0.18 (0.07, 0.43)	Lenva	0.31 (0.07, 1.32)	0.89 (0.19, 3.90)	0.71 (0.15, 3.20)	0.84(0.19, 3.6)	0.26 (0.08, 0.87)
PFS	Time-trend analysis	SUCRA(%)	15.26	75.02	35.65	65.94	64.38	57.45	36.27
		Pla	Pla	5.12 (1.21, 24.52)	1.60 (0.23, 12.64)	6.48 (0.01, 4124.67)	3.81 (0.55, 26.61)	3.6 (0.03, 329.40)	1.63 (0.23, 10.62)
		Lenva	0.19 (0.04, 0.82)	Lenva	0.31 (0.031, 3.19)	1.25 (0.00, 1694.51)	0.74 (0.06, 8.05)	0.681(0.01, 45.57)	0.31 (0.02, 3.62)
OS	Fixed-time-point analysis	SUCRA(%)	23.46	39.54	32.52	62.58	76.64	65.26	/
		Pla	Pla	1.19 (0.33, 4.36)	1.08 (0.36, 3.26)	1.76 (0.51, 6.08)	2.37 (0.63, 8.97)	1.85 (0.53, 6.47)	/
		Lenva	0.84 (0.23, 3.04)	Lenva	0.91 (0.17, 4.91)	1.48 (0.25, 8.81)	1.99 (0.31, 12.63)	1.56 (0.26, 9.41)	/
OS	Time-trend analysis	SUCRA(%)	29.94	42.51	36.65	56.59	73.99	60.32	/
		Pla	Pla	1.19 (0.26, 5.55)	1.08 (0.31, 3.75)	1.76 (0.01, 258.11)	2.38 (0.63, 9.06)	1.84 (0.05, 55.22)	/
		Lenva	0.84 (0.18, 3.87)	Lenva	0.9 (0.16, 5.03)	1.48 (0, 559.6)	2 (0.27, 14.75)	1.55 (0.08, 29.38)	/

Anlotinib (Anlo), apatinib (Apa), cabozantinib (Cabo), lenvatinib (Lenva), nintedanib (Ninted), sorafenib (Sora), vandetanib (Vande), and placebo (Pla). “Time-trend analysis” models landmark time points as a continuous covariate to explore how treatment effects evolve over time.

**Table 5:** Matrices of pairwise regimen comparisons for ORR and AEs from Bayesian network meta-analysis and time-trend modeling (reported as odds ratios with 95% credible intervals).

Outcomes	Methods	Control group	Pla	Lenva	Sora	Anlo	Apa	Cabo	Vande	Ninted
ORR	Fixed-time-point analysis	SUCRA(%)	14.91	82.09	59.81	75.55	68.11	49.65	32.69	17.20
		Pla	Pla	143.18 (4.18,5863.95)	27.99 (0.18,4089.41)	105.18 (0.49, 23384.12)	53.72 (0.34, 8363.00)	12.40 (0.05, 2523.73)	2.97 (0.11, 149.52)	0.56 (0, 212.61)
		Lenva	0.01 (0, 0.23)	Lenva	0.19 (0, 85.09)	0.72 (0., 441.27)	0.37 (0.001, 163.18)	0.08 (0, 47.85)	0.02 (0, 4.32)	0.01 (0, 3.74)
ORR	Time-trend analysis	SUCRA(%)	18.82	75.16	55.57	63.87	63.75	45.12	27.71	/
		Pla	Pla	134.99 (1.37,15463.66)	25.18 (0.04,14399.22)	180.64 (0,507327095493.77)	52.72 (0.14, 19322.53)	8.32 (0, 9027885.68)	1.64 (0.01, 546.30)	/
		Lenva	0.01 (0, 0.72)	Lenva	0.18 (0, 249.26)	1.27 (0, 35253744076.37)	0.39 (0, 639.99)	0.05 (0, 22661.25)	0.01 (0, 23.76)	/
AEs	Fixed-time-point analysis	Pla	Lenva	Sora	Anlo	Apa	Cabo	Vande	Ninted
		SUCRA(%)	56.45	26.36	37.66	36.47	93.16	47.35	58.57	41.56
		Pla	Pla	2.88 (0.28, 27.43)	1.88 (0.07, 45.54)	2.12 (0.07, 64.14)	0.07 (0, 2.54)	1.28 (0.05, 33.54)	0.85 (0.08, 8.53)	1.69 (0.06, 46.45)
		Lenva	0.34 (0.03, 3.4)	Lenva	0.64 (0.01, 34.68)	0.73 (0.01, 43.45)	0.02 (0, 1.52)	0.44 (0.01, 24.03)	0.30 (0.01, 7.85)	0.58 (0.01, 34.06)
AEs	Time-trend analysis	SUCRA(%)	56.81	28.82	38.54	57.24	67.24	36.36	44.28	/
		Pla	Pla	3.84 (0.23, 47.42)	2.61 (0.06, 72.89)	0.53 (0, 20776.62)	0.07 (0, 2.12)	3.56 (0, 210326.51)	1.59 (0.06, 50.93)	/
		Lenva	0.26 (0.021, 4.32)	Lenva	0.66 (0.01, 34.92)	0.15 (0, 23427.37)	0.01 (0, 1.62)	1.16 (0, 12371.71)	0.41 (0.01, 46.32)

Anlotinib (Anlo), apatinib (Apa), cabozantinib (Cabo), lenvatinib (Lenva), nintedanib (Ninted), sorafenib (Sora), vandetanib (Vande), and placebo (Pla). “Time-trend analysis” models landmark time points as a continuous covariate to explore how treatment effects evolve over time.

In the PFS analysis, compared to placebo, lenvatinib, anlotinib, apatinib, and cabozantinib demonstrated significant advantages, with hazards ratios (HR = 5.36, 95% CrI: 2.32–12.6), (HR = 4.78, 95% CrI: 1.38–16.40), (HR = 3.85, 95% CrI: 1.11–13.49), and (HR = 4.54, 95% CrI: 1.351–15.185), respectively. When lenvatinib was used as the reference, no significant differences were found among lenvatinib, anlotinib, apatinib, and cabozantinib. In the SUCRA ranking, lenvatinib ranked first with a score of 81.97%, followed by anlotinib (74.46%) and cabozantinib (71.91%).

In the analysis of OS, no drug showed better efficacy than placebo. Apatinib ranked first in the SUCRA ranking with a score of 76.64%.

In terms of ORR, lenvatinib demonstrated a significant advantage over placebo, with an OR = 143.18 (95% CrI: 4.18–5863.95). Lenvatinib also ranked first in the SUCRA ranking with a score of 82.09%.

Although none of the drugs differed significantly from placebo in terms of grade ≥3 adverse events, apatinib ranked highest in safety (SUCRA = 93.16%).

### 3.5 Network time-trend analysis

Network time-trend analysis was performed with the median follow-up time as a covariant. The corresponding network plot is shown in **Supplementary Figure S4B**. The results of direct and indirect comparisons at each time point are presented in **Tables 4**, **5** and **Supplementary Tables S3**-**S6**.

In the PFS time-trend analysis, lenvatinib showed significant preponderance over placebo (HR = 5.12, 95% CrI: 1.21 to 24.52), while other targeted therapies showed no significant differences. Lenvatinib ranked first in SUCRA (75.02%).

We plotted time-effect curves for each drug, with the y-axis indicating pairwise effect size. Results showed lenvatinib’s was superior to placebo between 13.84 and 18.15 months (**Supplementary Figure S5**).

In the OS time-trend analysis, no drug showed a significant difference compared to placebo.

For ORR, time-trend analysis, lenvatinib showed significant preponderance over placebo (OR = 134.99, 95% CrI: 1.37 to15463.66), while other targeted therapies showed no significant differences. Lenvatinib ranked first in SUCRA (75.16%).

In the AEs time-trend analysis, no drug showed a significant difference versus placebo. Apatinib had the lowest SUCRA value (67.24%), suggesting a better safety profile.

Time-effect curves were plotted for OS, ORR, and AEs, but no drug showed a significant time versus effect-size correlation (**Supplementary Figures S6**-**S8**).

The Brooks-Gelman-Rubin diagnostic confirmed stable and reproducible iterations for each Markov chain Monte Carlo (MCMC). Model convergence across all outcomes was verified using trace plots. Consistency was validated across pairwise comparisons in each endpoint. Detailed results are shown in **Supplementary Figures S9**-**12**. Inconsistency assessment for the primary network meta-analyses showed no significant global inconsistency: ΔDIC = 0.02 (ORR), 0.02 (PFS), 0.01 (OS), and 0.08 (grade ≥3 AEs). Additional analyses based on landmark time points (e.g., PFS at 3, 6, and 9 months) yielded similarly low ΔDIC values, further supporting the plausibility of the consistency assumption.

## 4 Discussion

To the best of our knowledge, this is the first Bayesian network time-trend analysis comprehensively evaluating the efficacy and safety of multiple targeted therapies for RAIR-DTC across multiple time points. Our study uniquely integrates data from various randomized controlled trials (RCTs) and employs Bayesian principles to explore the time-dependent effects of treatments, providing a more nuanced understanding of therapeutic outcomes.

Our findings indicate that lenvatinib, anlotinib, apatinib, and cabozantinib significantly improve progression-free survival (PFS) compared to placebo. Among these, lenvatinib demonstrated the most consistent benefit, maintaining a statistically significant PFS advantage throughout the 24-month observation period and achieving the highest SUCRA rankings across time points. In contrast, apatinib showed early efficacy as early as 3 months, but its effect appeared to attenuate by 6–9 months. These distinct temporal patterns—revealed through our time-trend modeling—suggest that the magnitude and durability of treatment effects vary across tyrosine kinase inhibitors (TKIs) in RAIR-DTC.

From a clinical perspective, these findings may inform the consideration of lenvatinib as a candidate for first-line therapy when sustained disease control, while apatinib’s early activity may position it as an initial option in a sequential strategy—potentially followed by lenvatinib upon early progression or stabilization. However, it is crucial to emphasize that these insights are derived from indirect network comparisons and exploratory time-trend analyses. The proposed sequencing approach remains hypothesis-generating and requires robust validation through prospective, head-to-head clinical trials before implementation in routine practice. Cabozantinib also demonstrated significant PFS improvements but did not maintain significance in the network time-trend analysis after adjustment for follow-up duration. This highlights the need for personalized treatment strategies based on individual patient characteristics and treatment timelines. Additionally, no targeted therapy showed significant improvements in OS compared to placebo, suggesting that further mechanistic studies are needed to elucidate the underlying reasons.

Lenvatinib’s significant advantage in progression-free survival (PFS) over placebo, particularly when it becomes evident after 6 months of treatment, may be closely related to its multi-targeted mechanism of action. Lenvatinib is a multi-targeted tyrosine kinase inhibitor that primarily inhibits tumor growth by targeting vascular endothelial growth factor receptors (VEGFR) and fibroblast growth factor receptors (FGFR) (24). Its inhibition of VEGFR1-3 and FGFR1-4 effectively blocks tumor angiogenesis, cutting off the “nutritional supply” to the tumor and thereby delaying tumor progression (25–27). Additionally, lenvatinib can inhibit other targets including RET, PDGFRα, and KIT, exerting precise anti-tumor effects on specific gene mutations (28, 29).

However, lenvatinib did not show a significant advantage in overall survival (OS), which may be attributed to resistance mechanisms. Studies have found that abnormal activation of the epidermal growth factor receptor (EGFR) (30), over-expression of FGFR1, and changes in the tumor micro-environment can all lead to resistance to lenvatinib (31). For instance, lenvatinib’s inhibition of FGFR may enhance the activity of the downstream MAPK signaling pathway of EGFR, leading to primary resistance (32). Moreover, overexpression of FGFR1 can induce the activation of the AKT/mTOR/ERK signaling pathway in HCC cells, resulting in lenvatinib resistance (30). The presence of these resistance mechanisms may limit lenvatinib’s improvement in OS, collectively.

Apatinib’s efficacy was only observed in the first 6 months, which may be related to its selective inhibition of VEGFR-2 (33). Apatinib blocks tumor angiogenesis by inhibiting VEGFR-2, but over time, tumor cells may activate other compensatory pathways to overcome this inhibition. Additionally, the pharmacokinetic properties of apatinib may also affect the duration of its efficacy. Its relatively rapid metabolism and excretion in the body may lead to a gradual decrease in drug concentration after 6 months, thereby weakening its inhibitory effect on tumors. Future research needs to further explore the mechanisms of action of apatinib in the treatment of RAIR-DTC and how to extend its efficacy through combination therapies or optimized dosing regimens.

Our study paved ways for future clinical research. Given the significant PFS benefits of lenvatinib, further exploration of its long-term safety and potential biomarkers of response is essential. Additionally, the transient efficacy of apatinib suggests the need for studies investigating combination therapies or sequential treatment regimens to overcome resistance mechanisms. Moreover, leveraging adverse event databases (i.e., FDA’s Adverse Event Reporting System (FAERS)) to gain a more precise understanding of each drug’s safety profile could inform personalized treatment strategies, minimizing toxicity while maximizing efficacy. Future research should also focus on identifying predictive biomarkers for treatment response and resistance, enabling more targeted therapeutic approaches for RAIR-DTC patients.

### 4.1 Limitation

Our study has several important limitations. First, the included randomized controlled trials were generally small in sample size and often reported low event counts for key outcomes (e.g., objective responses or grade ≥3 adverse events), leading to imprecise effect estimates with wide credible intervals. This limits both the precision and generalizability of our findings, and warrants cautious interpretation—particularly for comparisons with extreme point estimates but high uncertainty. Second, the overall number of studies was limited, and several treatment pairs lacked direct head-to-head comparisons, increasing reliance on indirect evidence and potentially amplifying uncertainty in the network estimates. Third, follow-up durations across trials were relatively short (ranging from 6.2 to 35.9 months), precluding a robust evaluation of long-term outcomes—particularly overall survival, which remains immature in most studies.

Furthermore, although we employed multiple landmark time points (e.g., 3-, 6-, and 12-month PFS rates) to explore temporal patterns in treatment effects, this approach does not fully capture the time-to-event nature of survival data. While each time point was analyzed in a separate network meta-analysis to preserve model independence, outcomes derived from the same trial across different time points remain inherently correlated. A formal time-to-event network meta-analysis based on hazard ratios would be methodologically superior; however, individual patient-level data or reconstructable Kaplan–Meier curves were unavailable for the majority of included studies. Consequently, our findings should be considered exploratory, and greater interpretive weight should be placed on effect size estimates—such as odds ratios or hazard ratios with their credible intervals—rather than on ranking probabilities (34).

## 5 Conclusion

Lenvatinib demonstrates the most consistent and durable PFS benefit among the TKIs evaluated for RAIR-DTC, which positions it as a leading candidate for first-line therapy. Apatinib shows early efficacy but waning effects beyond 6–9 months, suggesting a potential niche in sequential strategies. These distinct time-dependent efficacy patterns provide a hypothesis-generating framework for optimizing treatment selection and sequencing, which requires prospective validation in future clinical trials.

## Data Availability

The original contributions presented in the study are included in the article/**Supplementary Material**. Further inquiries can be directed to the corresponding authors.

## Supplementary material

Supplementary Figure 1 **(A)**. Risk of bias percentage assessment across different domains. **(B)**. Detailed Risk of Bias Assessment for Each Included Study.

Supplementary Figure 2 Network graphs of pairwise comparisons of treatment regimens at each time point of PFS. Anlotinib (Anlo), apatinib (Apa), cabozantinib (Cabo), lenvatinib (Lenva), nintedanib (Ninted), sorafenib (Sora), vandetanib (Vande), and placebo (Pla). The edges between nodes indicate the presence of direct comparisons between the regimens, with the thickness of the edges reflecting the number of studies contributing to each pairwise comparison.

Supplementary Figure 3 Network graphs of pairwise comparisons of treatment regimens at each time point of OS. Anlotinib (Anlo), apatinib (Apa), cabozantinib (Cabo), lenvatinib (Lenva), sorafenib (Sora), vandetanib (Vande), and placebo (Pla). The edges between nodes indicate the presence of direct comparisons between the regimens, with the thickness of the edges reflecting the number of studies contributing to each pairwise comparison.

Supplementary Figure 4 Network graphs comparing treatment regimens in terms of PFS, OS, ORR, and AEs. **(A)** Network graph derived from network meta-analysis. **(B)** Network graph derived from network regression meta-analysis. Each node represents a treatment regimen, and the edges between nodes indicate the presence of direct comparisons, with the thickness of the edges reflecting the number of studies contributing to each pairwise comparison. Anlotinib (Anlo), apatinib (Apa), cabozantinib (Cabo), lenvatinib (Lenva), nintedanib (Ninted), placebo (Pla), sorafenib (Sora), and vandetanib (Vande).

Supplementary Figure 5 Time-effect relationship curves showing PFS metric for various targeted therapies versus placebo.

Supplementary Figure 6 Time-effect relationship curves showing OS metric for various targeted therapies versus placebo.

Supplementary Figure 7 Time-effect relationship curves showing ORR metric for various targeted therapies versus placebo. Anlotinib (Anlo), apatinib (Apa), cabozantinib (Cabo), lenvatinib (Lenva), placebo (Pla), sorafenib (Sora), and vandetanib (Vande).

Supplementary Figure 8 Time-effect relationship curves showing AEs metric for various targeted therapies versus placebo. Anlotinib (Anlo), apatinib (Apa), cabozantinib (Cabo), lenvatinib (Lenva), placebo (Pla), sorafenib (Sora), and vandetanib (Vande).

Supplementary Figure 9 Brooks-Gelman-Rubin diagnostic convergence plots at each time point on PFS. **(A)** 3 months, **(B)** 6 months, **(C)** 9 months, **(D)** 12 months, **(E)** 18 months, and **(F)** 24 months. Placebo (Pla), lenvatinib (Lenva), sorafenib (Sora), anlotinib (Anlo), apatinib (Apa), cabozantinib (Cabo), vandetanib (Vande), and nintedanib (Ninted).

Supplementary Figure 10 Brooks-Gelman-Rubin diagnostic convergence plots at each time point on OS. **(A)** 3 months, **(B)** 6 months, **(C)** 9 months, **(D)** 12 months, **(E)** 18 months, and **(F)** 24 months. Anlotinib (Anlo), apatinib (Apa), cabozantinib (Cabo), placebo (Pla), lenvatinib (Lenva), sorafenib (Sora), and vandetanib (Vande).

Supplementary Figure 11 Brooks-Gelman-Rubin diagnostic convergence plots on PFS, OS, ORR, AEs. **(A)** PFS, **(B)** OS, **(C)** ORR, and **(D)** AEs. Anlotinib (Anlo), apatinib (Apa), cabozantinib (Cabo), placebo (Pla), lenvatinib (Lenva), sorafenib (Sora), vandetanib (Vande), and nintedanib (Ninted).

Supplementary Figure 12 Brooks-Gelman-Rubin diagnostic convergence plots on PFS, OS, ORR, AEs (regression meta-analysis). **(A)** PFS, **(B)** OS, **(C)** ORR, **(D)** AEs. Anlotinib (Anlo), apatinib (Apa), cabozantinib (Cabo), placebo (Pla), lenvatinib (Lenva), sorafenib (Sora), vandetanib (Vande), and nintedanib (Ninted).

Supplementary Table 1 Matrices of pairwise comparisons of regimens for PFS at each time point from Bayesian network meta-analysis (presented as odds ratios and 95% credible intervals).

Supplementary Table 2 Matrices of pairwise comparisons of regimens for OS at each time point from Bayesian network meta-analysis (presented as odds ratios and 95% credible intervals).

Supplementary Table 3 Matrices of pairwise comparisons of regimens for PFS from Bayesian network meta-analysis (presented as hazard ratios and 95% credible intervals).

Supplementary Table 4 Matrices of pairwise comparisons of regimens for OS from Bayesian network meta-analysis (presented as hazard ratios and 95% credible intervals).

Supplementary Table 5 Matrices of pairwise comparisons of regimens for ORR from Bayesian network meta-analysis (shown as odds ratios and 95% confidence intervals).

Supplementary Table 6 Matrices of pairwise comparisons of regimens for AEs from Bayesian network meta-analysis (shown as odds ratios and 95% confidence intervals).
