# Supplementary figures and images for: A Bayesian network meta-analysis: evaluating the efficacy and safety of targeted therapies in metastatic or advanced radioiodine-refractory differentiated thyroid cancer
Source: Front Oncol. 2026 Feb 27;16:1720670. doi: 10.3389/fonc.2026.1720670 (PMC12982094; doi:10.3389/fonc.2026.1720670)

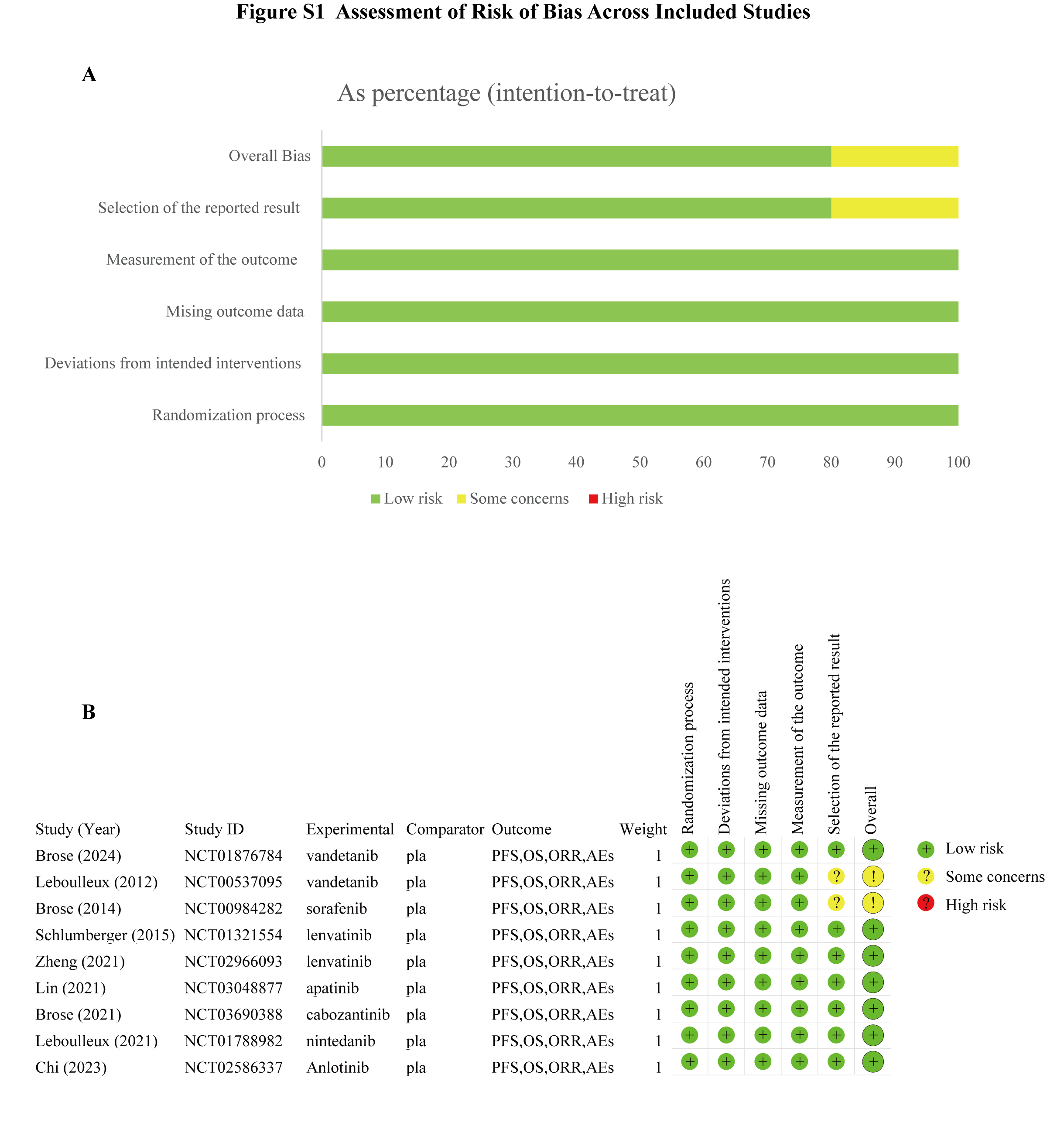

Supplement: Supplementary Figure 1 — (A). Risk of bias percentage assessment across different domains. (B). Detailed Risk of Bias Assessment for Each Included Study. [file DataSheet1.zip › figure S1.tif]

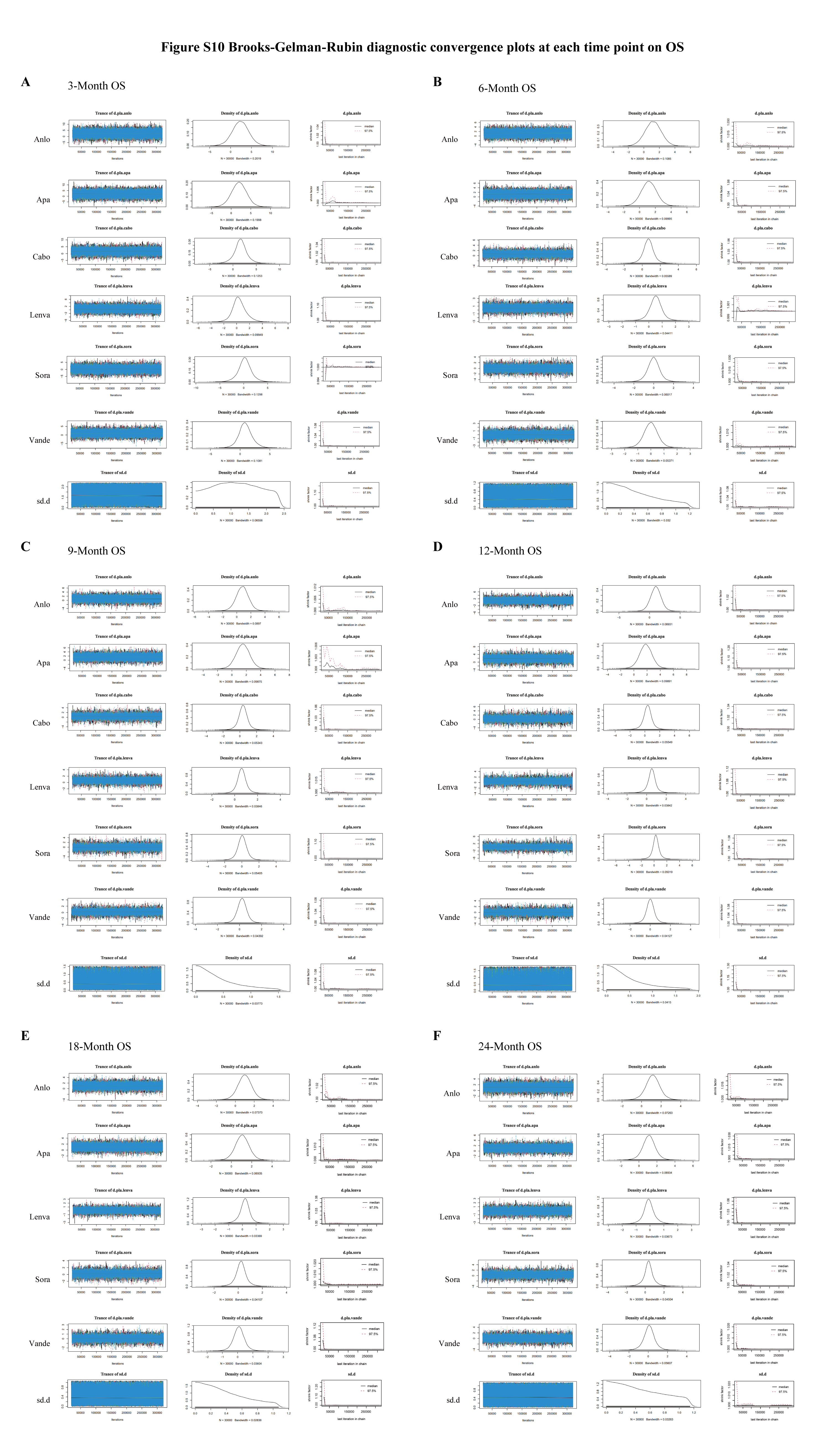

Supplement: Supplementary Figure 1 — (A). Risk of bias percentage assessment across different domains. (B). Detailed Risk of Bias Assessment for Each Included Study. [file DataSheet1.zip › figure S10.tif]

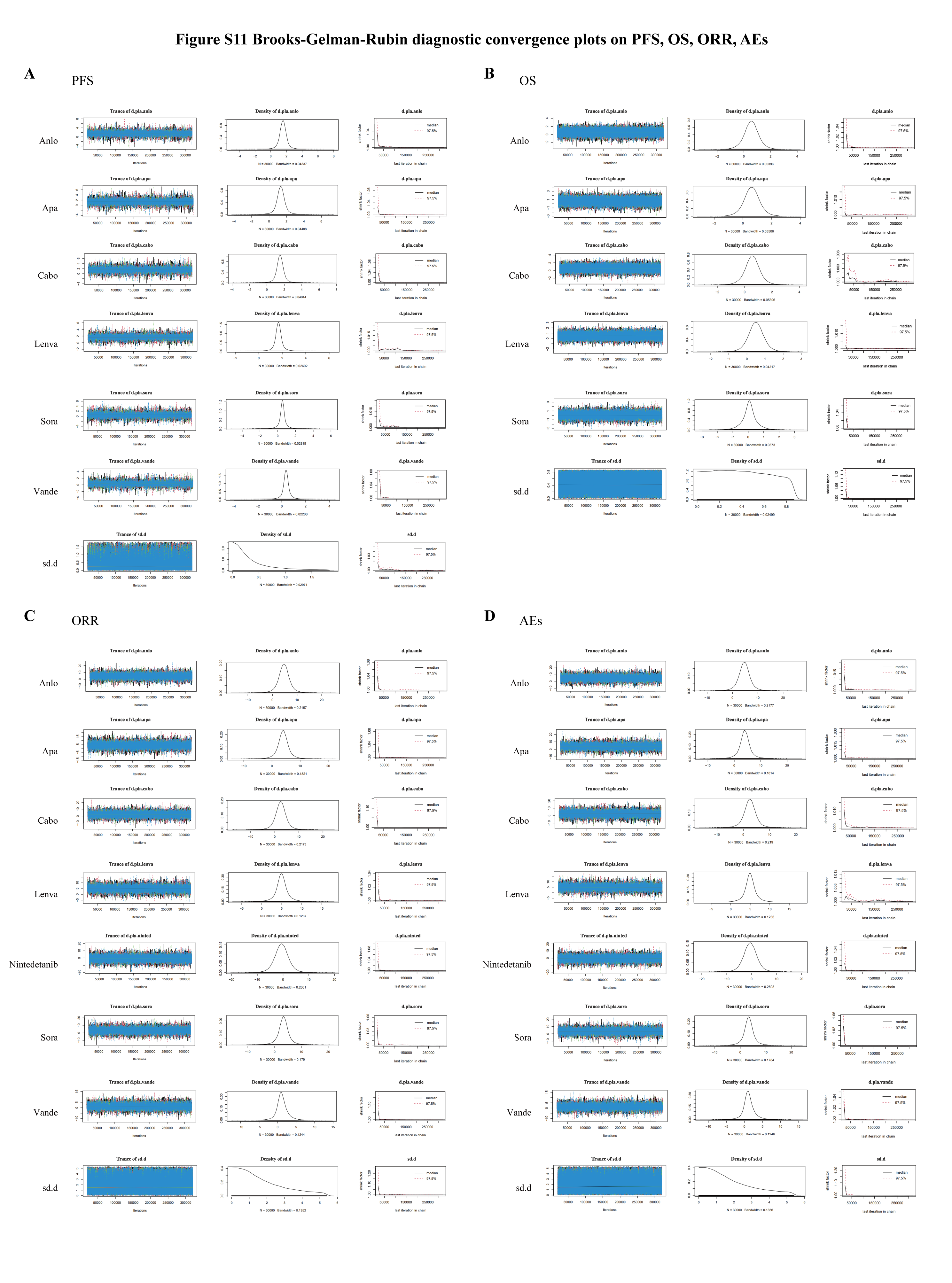

Supplement: Supplementary Figure 1 — (A). Risk of bias percentage assessment across different domains. (B). Detailed Risk of Bias Assessment for Each Included Study. [file DataSheet1.zip › figure S11.tif]

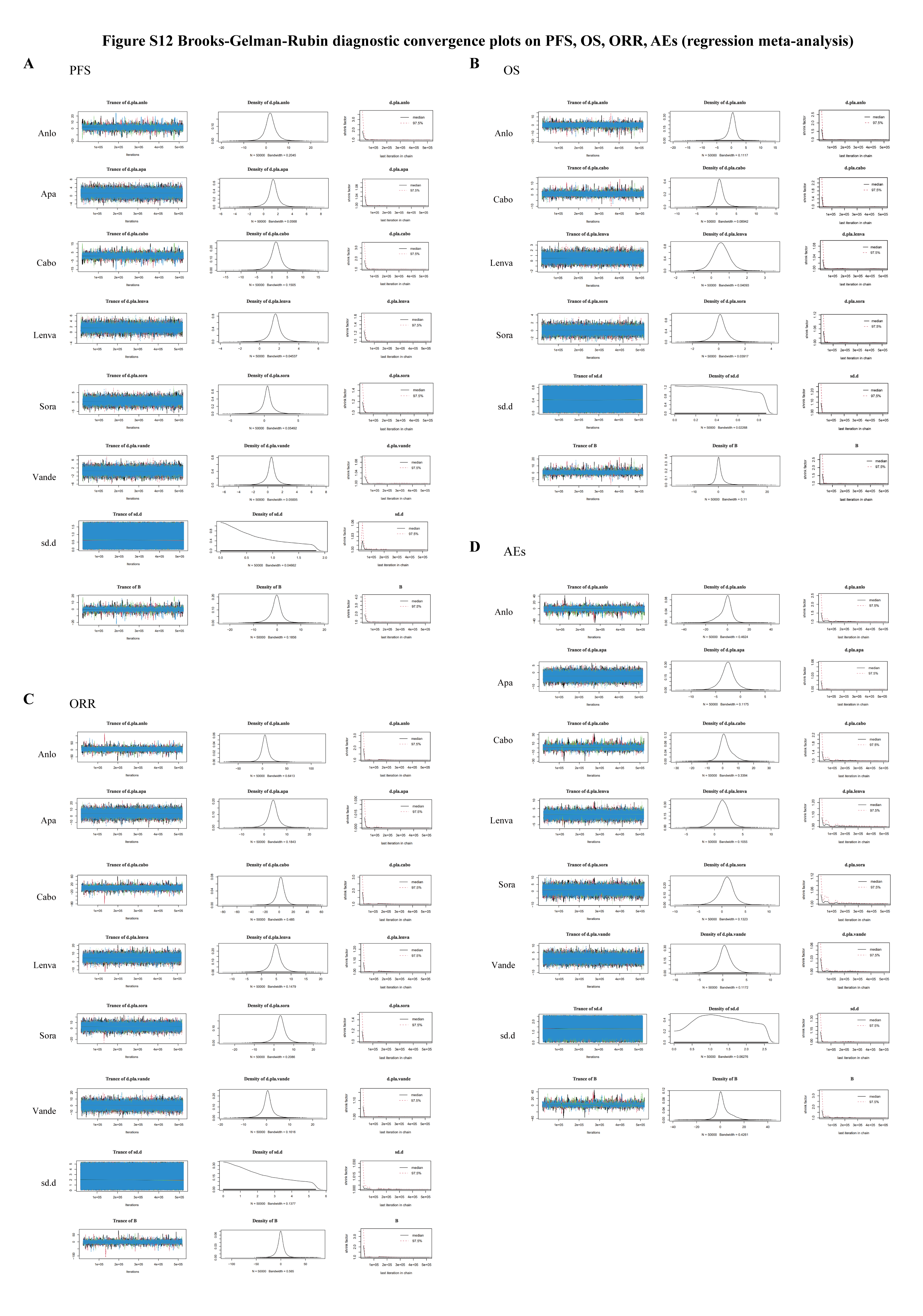

Supplement: Supplementary Figure 1 — (A). Risk of bias percentage assessment across different domains. (B). Detailed Risk of Bias Assessment for Each Included Study. [file DataSheet1.zip › figure S12.tif]

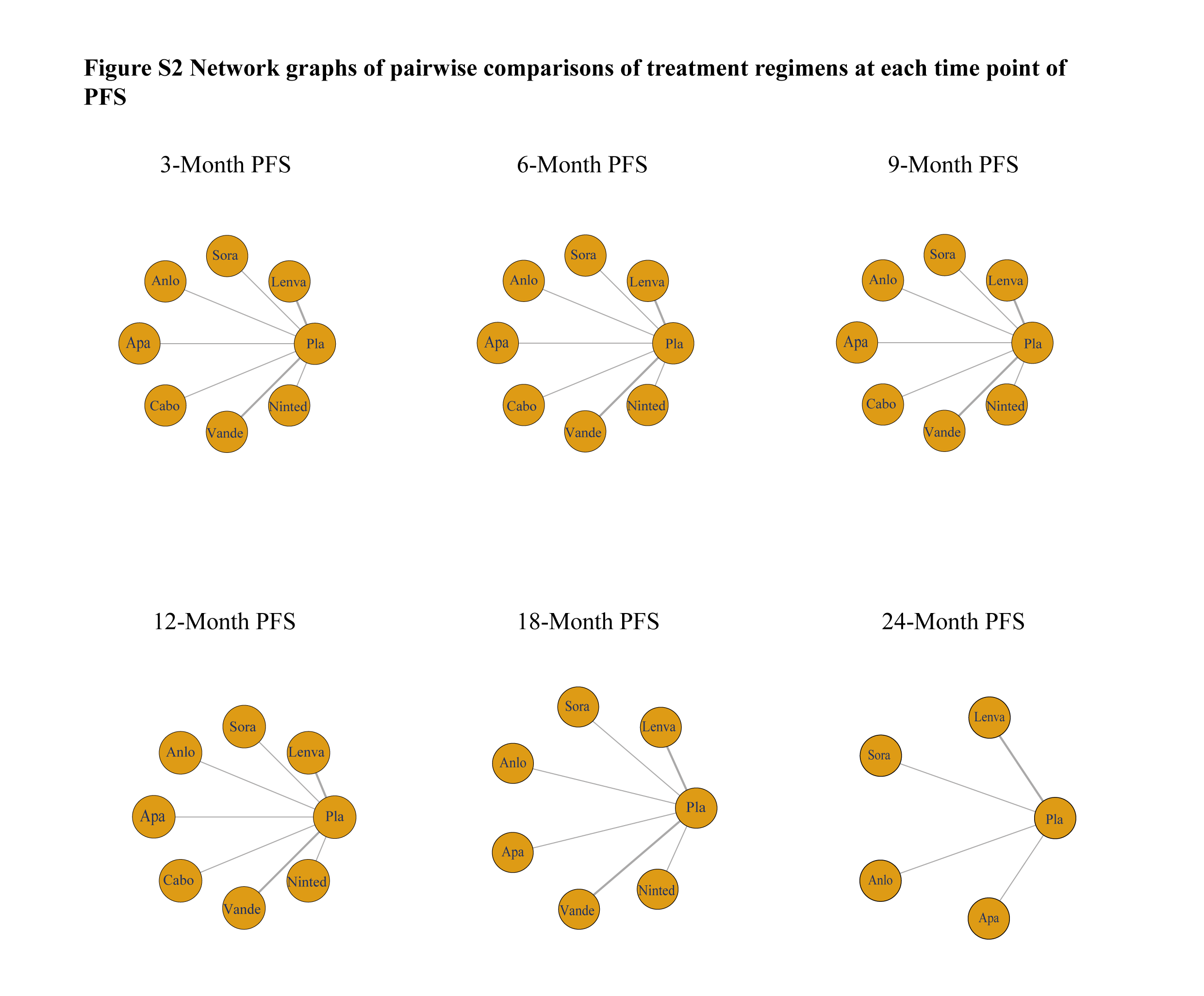

Supplement: Supplementary Figure 1 — (A). Risk of bias percentage assessment across different domains. (B). Detailed Risk of Bias Assessment for Each Included Study. [file DataSheet1.zip › figure S2.tif]

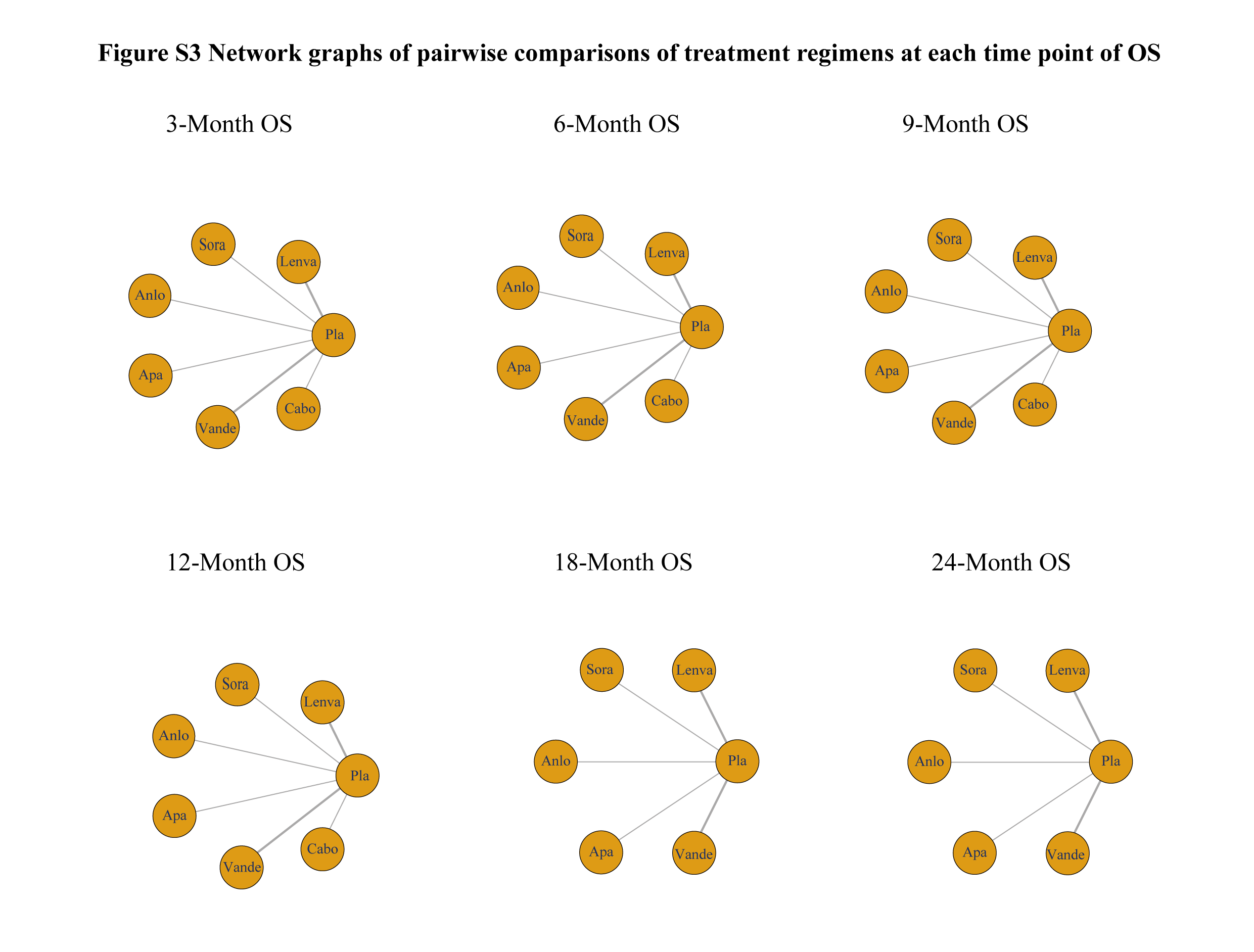

Supplement: Supplementary Figure 1 — (A). Risk of bias percentage assessment across different domains. (B). Detailed Risk of Bias Assessment for Each Included Study. [file DataSheet1.zip › figure S3.tif]

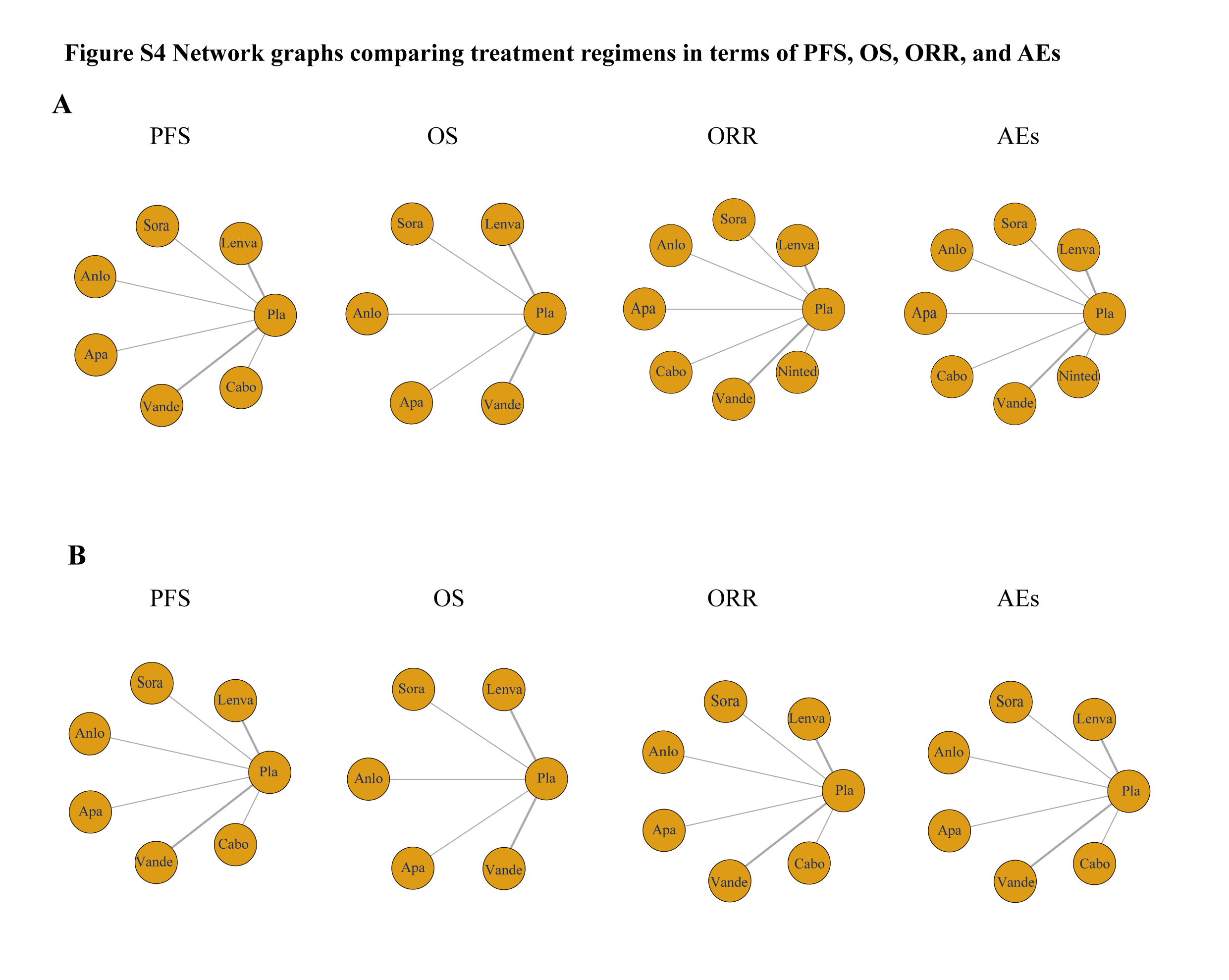

Supplement: Supplementary Figure 1 — (A). Risk of bias percentage assessment across different domains. (B). Detailed Risk of Bias Assessment for Each Included Study. [file DataSheet1.zip › figure S4.tif]

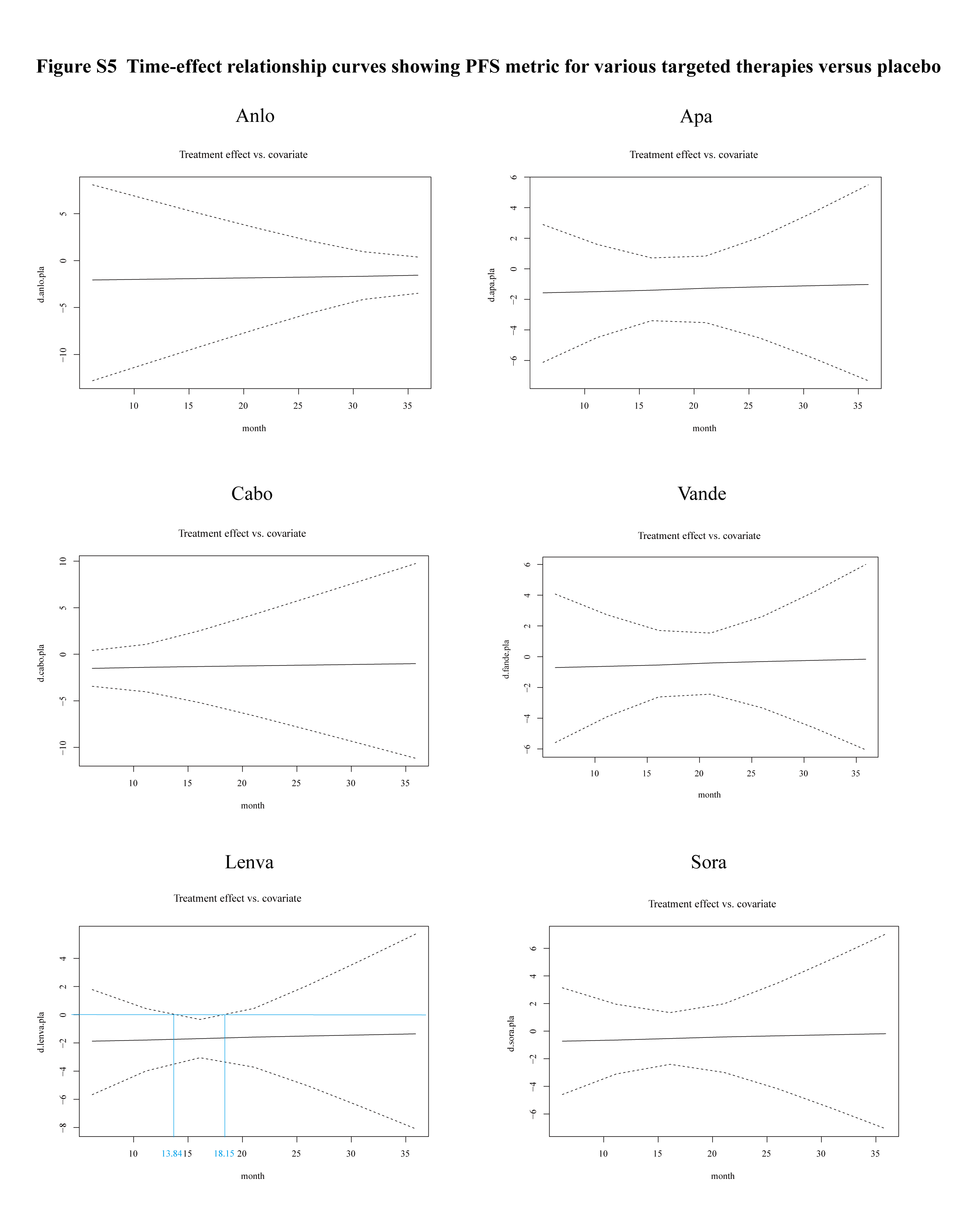

Supplement: Supplementary Figure 1 — (A). Risk of bias percentage assessment across different domains. (B). Detailed Risk of Bias Assessment for Each Included Study. [file DataSheet1.zip › figure S5.tif]

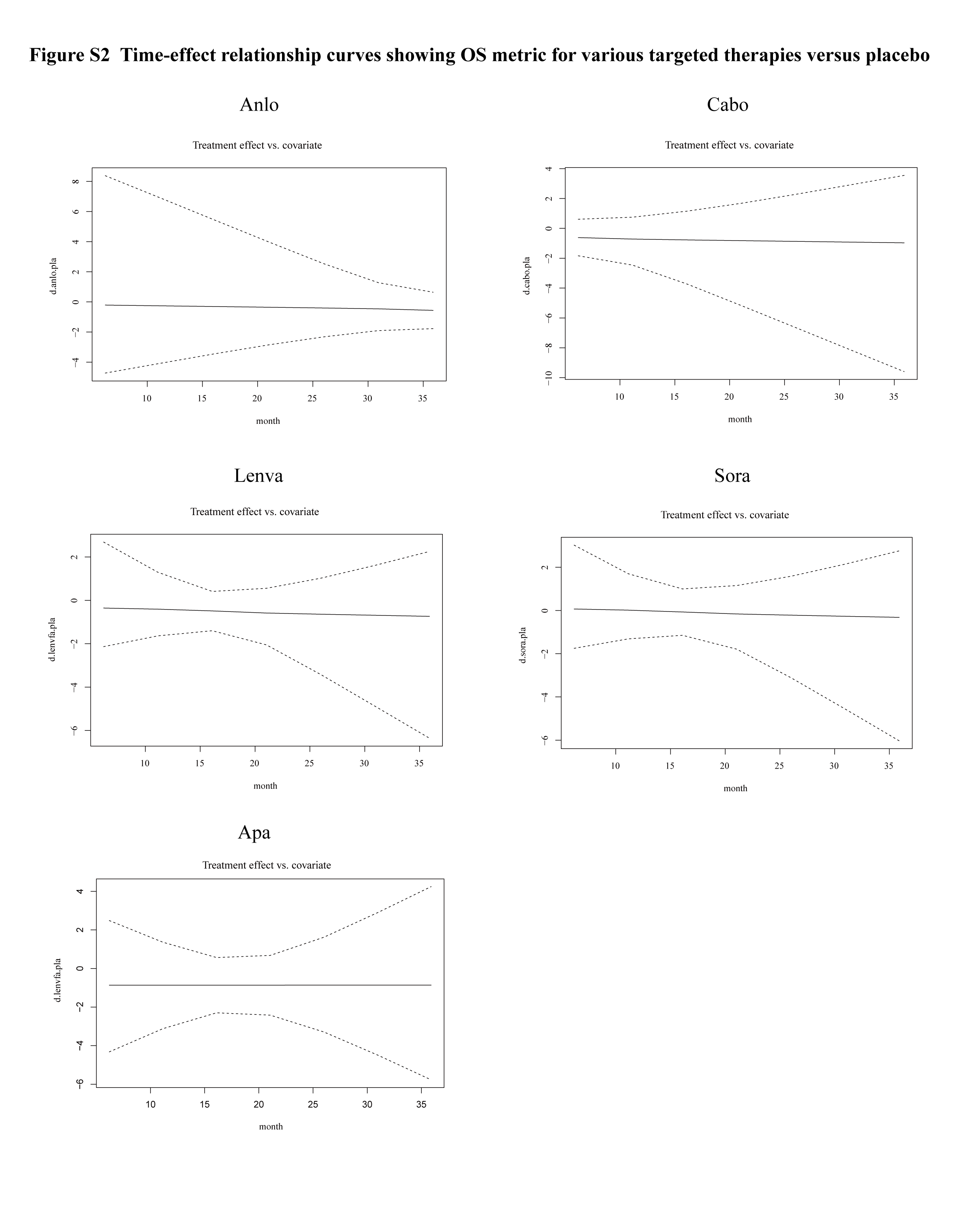

Supplement: Supplementary Figure 1 — (A). Risk of bias percentage assessment across different domains. (B). Detailed Risk of Bias Assessment for Each Included Study. [file DataSheet1.zip › figure S6.tif]

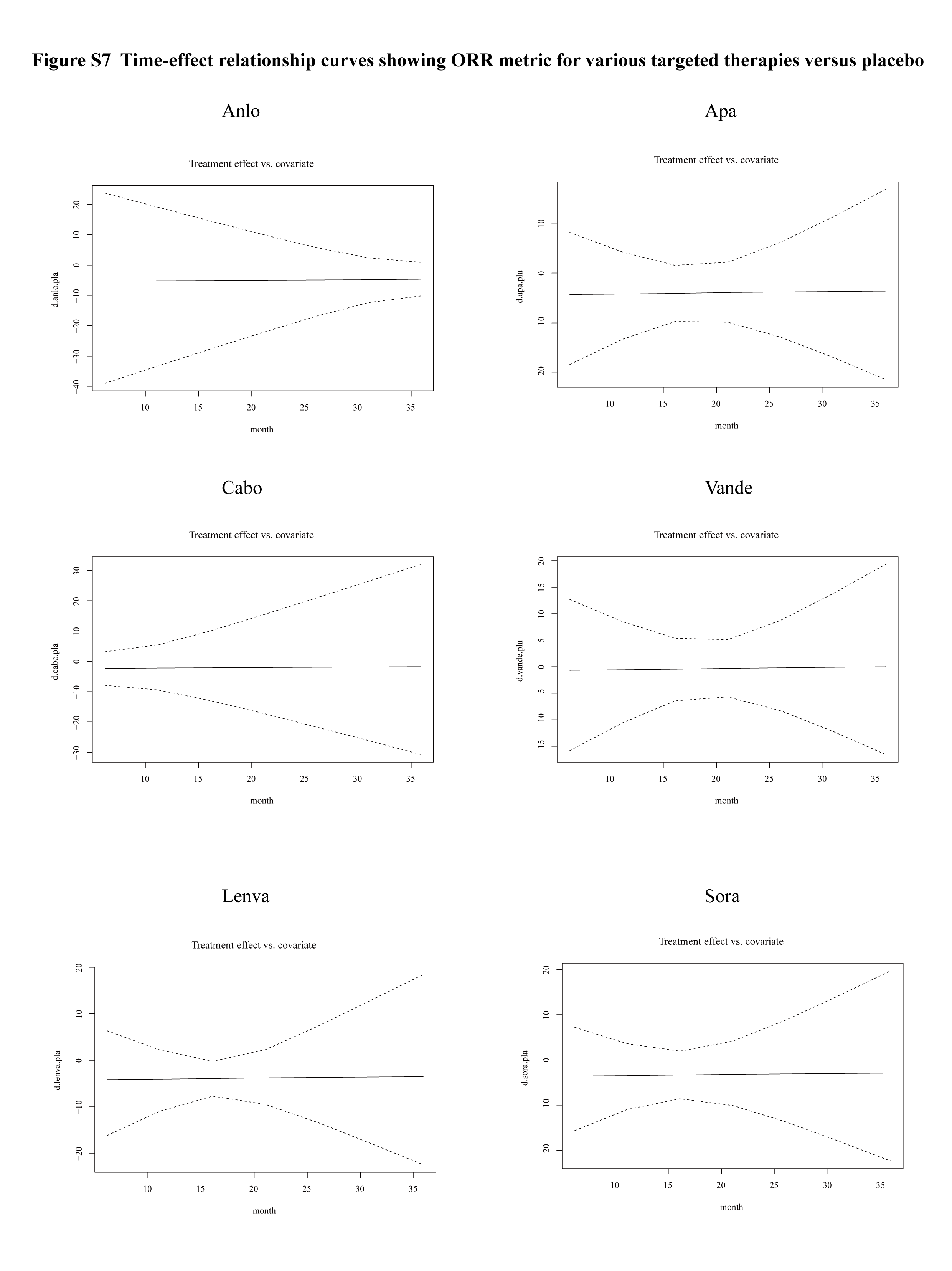

Supplement: Supplementary Figure 1 — (A). Risk of bias percentage assessment across different domains. (B). Detailed Risk of Bias Assessment for Each Included Study. [file DataSheet1.zip › figure S7.tif]

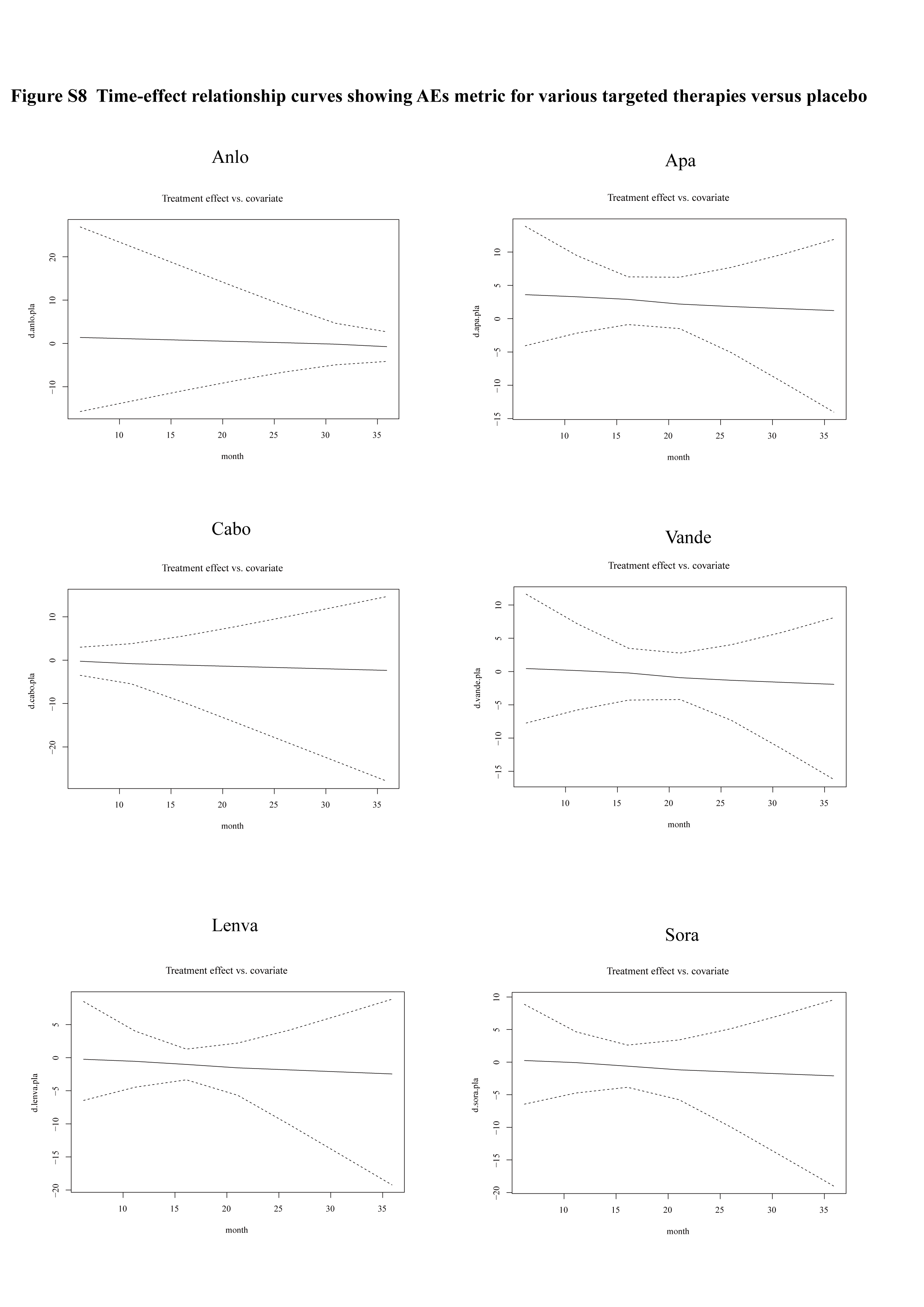

Supplement: Supplementary Figure 1 — (A). Risk of bias percentage assessment across different domains. (B). Detailed Risk of Bias Assessment for Each Included Study. [file DataSheet1.zip › figure S8.tif]

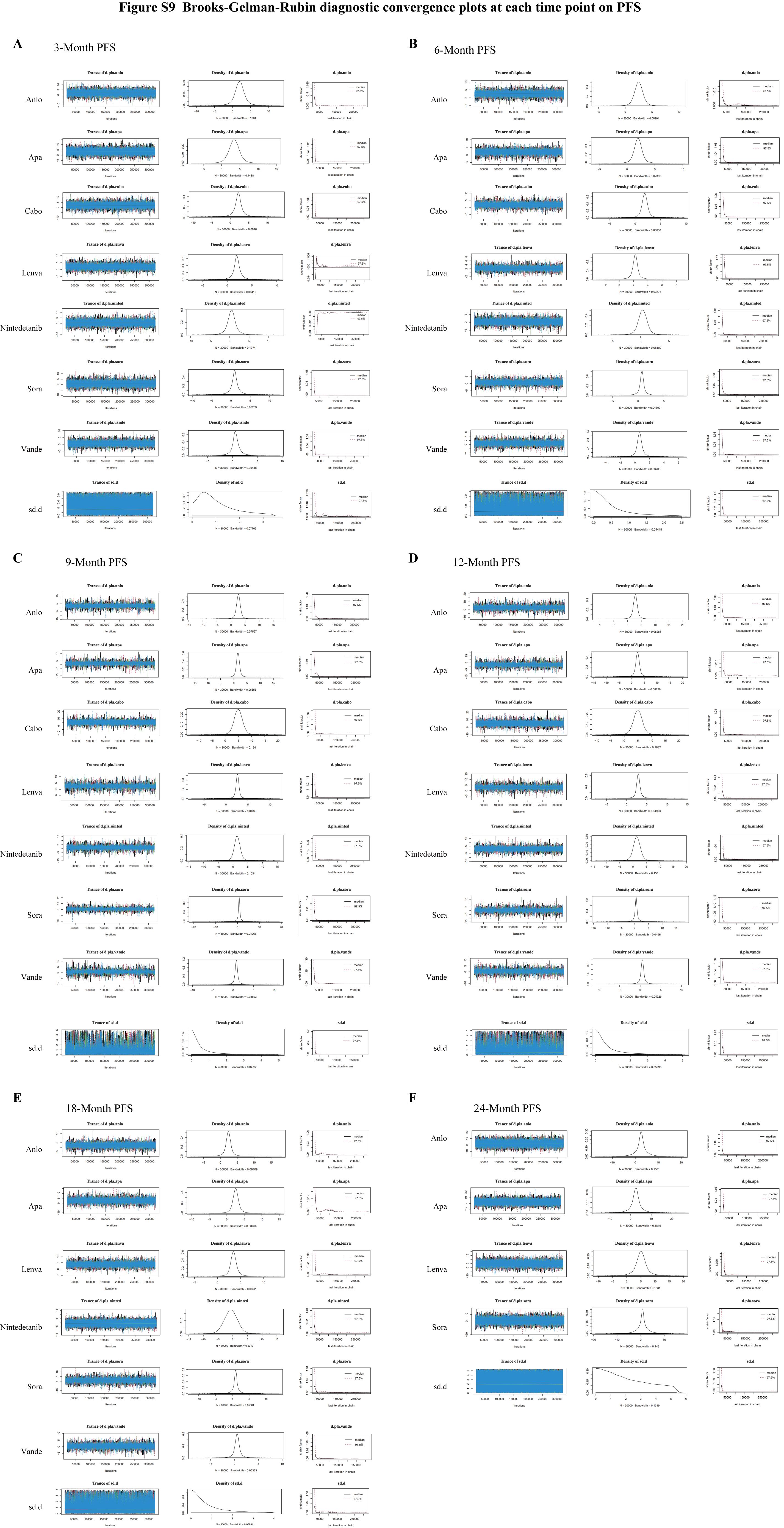

Supplement: Supplementary Figure 1 — (A). Risk of bias percentage assessment across different domains. (B). Detailed Risk of Bias Assessment for Each Included Study. [file DataSheet1.zip › figure S9.tif]
