# Supplementary material for: A Bayesian network meta-analysis: evaluating the efficacy and safety of targeted therapies in metastatic or advanced radioiodine-refractory differentiated thyroid cancer
Source: Front Oncol. 2026 Feb 27;16:1720670. doi: 10.3389/fonc.2026.1720670 (PMC12982094; doi:10.3389/fonc.2026.1720670)
Supplement: Supplementary file 9 [file DataSheet2.docx]

Supplementary File S2. Detailed search strategies for each database

| **Database** | **Search Terms** | **Filters** | **Date Range** |
| --- | --- | --- | --- |
| PubMed | (thyroid cancer OR differentiated thyroid cancer OR radioactive iodine-resistant thyroid cancer OR RAIR-DTC OR metastatic differentiated thyroid cancer OR advanced differentiated thyroid cancer) AND (targeted therapy OR tyrosine kinase inhibitor OR TKI OR VEGFR inhibitor OR FGFR inhibitor OR RET inhibitor OR lenvatinib OR sorafenib OR cabozantinib OR anlotinib OR apatinib) | Humans, Clinical Trial | From inception to July 29, 2025 |
| Embase | ('thyroid neoplasm'/exp OR 'thyroid cancer' OR 'differentiated thyroid cancer' OR 'radioactive iodine-resistant thyroid cancer' OR 'RAIR-DTC' OR 'metastatic differentiated thyroid cancer' OR 'advanced differentiated thyroid cancer') AND ('targeted therapy' OR 'tyrosine kinase inhibitor' OR 'TKI' OR 'VEGFR inhibitor' OR 'FGFR inhibitor' OR 'RET inhibitor' OR 'lenvatinib' OR 'sorafenib' OR 'cabozantinib' OR 'anlotinib' OR 'apatinib') | Humans, Clinical Trial | From inception to July 29, 2025 |
| Cochrane Library | (MeSH: "Thyroid Neoplasms" OR "Thyroid Cancer" OR "Differentiated Thyroid Cancer" OR "Radioactive Iodine-Resistant Thyroid Cancer" OR "RAIR-DTC" OR "Metastatic Differentiated Thyroid Cancer" OR "Advanced Differentiated Thyroid Cancer") AND (MeSH: "Targeted Therapy" OR "Tyrosine Kinase Inhibitor" OR "TKI" OR "VEGFR Inhibitor" OR "FGFR Inhibitor" OR "RET Inhibitor" OR "Lenvatinib" OR "Sorafenib" OR "Cabozantinib" OR "Anlotinib" OR "Apatinib") | Humans, Clinical Trial | From inception to July 29, 2025 |
| Scopus | ("thyroid cancer" OR "differentiated thyroid cancer" OR "radioactive iodine-resistant thyroid cancer" OR "RAIR-DTC" OR "metastatic differentiated thyroid cancer" OR "advanced differentiated thyroid cancer") AND ("targeted therapy" OR "tyrosine kinase inhibitor" OR "TKI" OR "VEGFR inhibitor" OR "FGFR inhibitor" OR "RET inhibitor" OR "lenvatinib" OR "sorafenib" OR "cabozantinib" OR "anlotinib" OR "apatinib") | Humans, Clinical Trial | From inception to July 29, 2025 |
| ClinicalTrials.gov | ("thyroid cancer" OR "differentiated thyroid cancer" OR "radioactive iodine-resistant thyroid cancer" OR "RAIR-DTC" OR "metastatic differentiated thyroid cancer" OR "advanced differentiated thyroid cancer") AND ("targeted therapy" OR "tyrosine kinase inhibitor" OR "TKI" OR "VEGFR inhibitor" OR "FGFR inhibitor" OR "RET inhibitor" OR "lenvatinib" OR "sorafenib" OR "cabozantinib" OR "anlotinib" OR "apatinib") | Humans, Clinical Trial | From inception to July 29, 2025 |
| Google Scholar | ("thyroid cancer" OR "differentiated thyroid cancer" OR "radioactive iodine-resistant thyroid cancer" OR "RAIR-DTC" OR "metastatic differentiated thyroid cancer" OR "advanced differentiated thyroid cancer") AND ("targeted therapy" OR "tyrosine kinase inhibitor" OR "TKI" OR "VEGFR inhibitor" OR "FGFR inhibitor" OR "RET inhibitor" OR "lenvatinib" OR "sorafenib" OR "cabozantinib" OR "anlotinib" OR "apatinib") | N/A | From inception to July 29, 2025 |
