# Supplementary material for: A Bayesian network meta-analysis: evaluating the efficacy and safety of targeted therapies in metastatic or advanced radioiodine-refractory differentiated thyroid cancer
Source: Front Oncol. 2026 Feb 27;16:1720670. doi: 10.3389/fonc.2026.1720670 (PMC12982094; doi:10.3389/fonc.2026.1720670)
Supplement: Supplementary file 10 [file DataSheet3.docx]

**Supplementary File S3. Annotated R code for Bayesian network meta-analyses and time-trend modeling**

**Supplementary File S3A. R code for HR-based network meta-analysis (PFS/OS)**

# Bayesian NMA for time-to-event outcomes using published hazard ratios (HRs)

# Outcome: Progression-free survival (PFS) or overall survival (OS)

# Input: lnHR and its standard error (SE)

library(gemtc)

library(coda)

# Load data: columns = study, treat1, treat2, lnHR, se_lnHR

hr_data <- read.csv("PFS_HR_data.csv")

# Build network

network_hr <- mtc.network(data.re = hr_data, studies = "study")

# Fit random-effects model on lnHR scale

model_hr <- mtc.model(network_hr,

likelihood = "normal", # continuous outcome (lnHR)

link = "identity",

linearModel = "random") # accounts for between-study heterogeneity

# Run MCMC: 300,000 iterations, 20,000 burn-in, thinning = 10

results_hr <- mtc.run(model_hr, n.adapt = 20000, n.iter = 300000, thin = 10)

# Convergence diagnostics

plot(results_hr) # trace, density, Brooks-Gelman-Rubin plots

# Extract results: HRs with 95% credible intervals

hr_effects <- relative.effect.table(results_hr)

sucra_hr <- sucra(results_hr)

# Save or print results

print(hr_effects)

**Supplementary File S3B. R code for OR-based network meta-analysis (ORR / adverse events)**

# Bayesian NMA for binary outcomes: objective response rate (ORR) or grade ≥3 AEs

# Input: number of events and total patients per treatment arm

library(gemtc)

library(coda)

# Load arm-level data: columns = study, treat, responders, total

bin_data <- read.csv("binary_outcome_data.csv")

# Build network from arm-level counts

network_bin <- mtc.network(data.ab = bin_data, treatments = "treat")

# Fit random-effects model on log-odds scale

model_bin <- mtc.model(network_bin,

likelihood = "binom", # binomial distribution

link = "logit", # log-odds (natural scale for OR)

linearModel = "random")

# Run MCMC with same settings as HR analysis

results_bin <- mtc.run(model_bin, n.adapt = 20000, n.iter = 300000, thin = 10)

# Convergence diagnostics

plot(results_bin)

# Extract results: odds ratios (ORs) with 95% CrIs

or_effects <- relative.effect.table(results_bin)

sucra_bin <- sucra(results_bin)

# Output results

print(or_effects)

**Supplementary File S3C. R code for time-trend modeling (exploratory analysis)**

library(gemtc)

library(coda)

library(ggplot2)

library(dplyr)

# -------------------------------------------------

# 1. Time-trend model for PFS (HR-based)

# Data must include: study, treat1, treat2, lnHR, se_lnHR, time_month

# -------------------------------------------------

hr_data <- read.csv("PFS_HR_with_time.csv")

net_hr <- mtc.network(data.re = hr_data, studies = "study")

model_hr_trend <- mtc.model(net_hr,

type = "regression",

regressor = list(coefficient = "shared", variable = "time_month"),

likelihood = "normal",

link = "identity",

linearModel = "random")

res_hr <- mtc.run(model_hr_trend, n.adapt = 20000, n.iter = 300000, thin = 10)

# -------------------------------------------------

# 2. Time-trend model for binary outcomes (e.g., ORR)

# Data must include: study, treat, responders, total, time_month

# -------------------------------------------------

bin_data <- read.csv("binary_with_time.csv")

net_bin <- mtc.network(data.ab = bin_data, treatments = "treat")

model_bin_trend <- mtc.model(net_bin,

type = "regression",

regressor = list(coefficient = "shared", variable = "time_month"),

likelihood = "binom",

link = "logit",

linearModel = "random")

res_bin <- mtc.run(model_bin_trend, n.adapt = 20000, n.iter = 300000, thin = 10)

# -------------------------------------------------

# 3. Extract and summarize time-dependent effects

# -------------------------------------------------

# Example: get HRs at 3, 6, 9, 12 months vs reference treatment

time_points <- c(3, 6, 9, 12)

hr_results <- lapply(time_points, function(t) {

df <- relative.effect.table(res_hr, covariate = t)

df$time_month <- t

return(df)

})

hr_long <- do.call(rbind, hr_results)

# Similarly for ORs (e.g., ORR at 3, 6, 9 months)

or_time_points <- c(3, 6, 9)

or_results <- lapply(or_time_points, function(t) {

df <- relative.effect.table(res_bin, covariate = t)

df$time_month <- t

return(df)

})

or_long <- do.call(rbind, or_results)

# -------------------------------------------------

# 4. Plot time-effect relationships

# -------------------------------------------------

# Plot HR over time (assuming 'A' is reference; adjust as needed)

pfs_plot <- ggplot(hr_long, aes(x = time_month, y = mean, color = comparison)) +

geom_line() +

geom_ribbon(aes(ymin = `2.5%`, ymax = `97.5%`), alpha = 0.2, color = NA) +

labs(title = "Time-Trend in PFS Treatment Effects (HR)",

x = "Follow-up Time (months)",

y = "Hazard Ratio (vs Reference)") +

theme_minimal()

# Plot OR over time (e.g., ORR)

orr_plot <- ggplot(or_long, aes(x = time_month, y = exp(mean), color = comparison)) +

geom_line() +

geom_ribbon(aes(ymin = exp(`2.5%`), ymax = exp(`97.5%`)), alpha = 0.2, color = NA) +

labs(title = "Time-Trend in ORR Treatment Effects (OR)",

x = "Assessment Time (months)",

y = "Odds Ratio (vs Reference)") +

theme_minimal()

# -------------------------------------------------

# 5. Save results and figures

# -------------------------------------------------

write.csv(hr_long, "time_trend_PFS_results.csv", row.names = FALSE)

write.csv(or_long, "time_trend_ORR_results.csv", row.names = FALSE)

ggsave("Figure_SX_PFS_time_trend.png", plot = pfs_plot, width = 8, height = 6, dpi = 300)

ggsave("Figure_SX_ORR_time_trend.png", plot = orr_plot, width = 8, height = 6, dpi = 300)

# Optional: display plots in R session

print(pfs_plot)

print(orr_plot)
